# Facing the challenges of PROM implementation in Dutch dialysis care: Patients’ and professionals’ perspectives

**DOI:** 10.1371/journal.pone.0285822

**Published:** 2023-05-15

**Authors:** Wim S. Sipma, Margriet F. C. de Jong, Yvette Meuleman, Marc H. Hemmelder, Kees (C.) T. B. Ahaus

**Affiliations:** 1 Department of Health Services Management & Organisation, Erasmus School of Health Policy & Management, Erasmus University Rotterdam, Rotterdam, The Netherlands; 2 Department of Nephrology, University Medical Centre Groningen, Groningen, The Netherlands; 3 Department of Clinical Epidemiology, Leiden University Medical Centre, Leiden, The Netherlands; 4 Department of Internal Medicine, Division of Nephrology, Maastricht University Medical Centre, Maastricht, The Netherlands; 5 CARIM school for cardiovascular research, University of Maastricht, Maastricht, The Netherlands; Sant’Anna School of Advanced Studies: Scuola Superiore Sant’Anna, ITALY

## Abstract

**Background:**

Patient Reported Outcome Measures (PROMs) are increasingly used in routine clinical practice to facilitate patients in sharing and discussing health-related topics with their clinician. This study focuses on the implementation experiences of healthcare professionals and patients during the early implementation phase of the newly developed Dutch set of dialysis PROMs and aims to understand the process of early implementation of PROMs from the users’ perspectives.

**Methods:**

This is a qualitative study among healthcare professionals (physicians and nursing staff: n = 13) and patients (n = 14) of which 12 were receiving haemodialysis and 2 peritoneal dialysis. Semi-structured interviews were used to understand the barriers and facilitators that both professionals and patients encounter when starting to implement PROMs.

**Results:**

The early PROM implementation process is influenced by a variety of factors that we divided into barriers and facilitators. We identified four barriers: patient´s indifference to PROMs, scepticism on the benefits of aggregated PROM data, the limited treatment options open to doctors and organizational issues such as mergers, organizational problems and renovations. We also describe four facilitators: professional involvement and patient support, a growing understanding of the use of PROMs during the implementation, quick gains from using PROMs such as receiving instant feedback and a clear ambition on patient care such as a shared view on patient involvement and management support.

**Conclusions:**

In this qualitative study carried out during the early implementation phase of the Dutch dialysis PROM set, we found that patients did not yet consider the PROM set to be a useful additional tool to share information with their doctor. This was despite the professionals’ primary reason for using PROMs being to improve patient–doctor communication. Furthermore, the perceived lack of intervention options was frustrating for some of the professionals. We found that nurses could be important enablers of further implementation because of their intensive relationship with dialysis patients.

## Introduction

Quality measures in healthcare have long been focused on care processes and clinical status such as objective outcomes (e.g. survival rates) and have historically been dominated by the perspectives of healthcare professionals [1,2]. However, these measurements only partially reflect the value of healthcare as perceived by patients. Patients are in particular interested in healthcare outcomes that matter to them personally such as functional status and quality of life [1]. To measure value of healthcare as perceived by patients, patient-reported outcome measures (PROMs) are increasingly used in routine clinical practice. PROMs are questionnaires that allow patients to systematically share their health-related quality of life scores and disease symptoms with their clinician. The use of PROMs has a number of potential benefits such as to deepen patient–clinician communication, engage patients in their treatment, help clinicians focus on patients’ needs, evaluate the effectiveness of interventions, improve overall healthcare quality and, as an overarching goal, contribute to better patient wellbeing [1–5]. The potential benefits of PROMs are widely recognized in healthcare literature for preventive care, primary care, incidental surgery, chronical diseases and palliative care [6–10].

However, these potential benefits are not easily achieved and are hindered by two issues. First, the implementation of PROMs in healthcare settings faces several barriers including patient and physician scepticism about practicability, time constraints, fear of added work, lack of training, administrative burden on patients and staff and a lack of organizational support [3,11–14] that result in a slow dissemination and use of PROMs. Also, sometimes patients do not complete PROMs because of simply forgetting or loss of motivation [15]. Second, there are doubts whether PROMs, once implemented, deliver the benefits they promise [12,16–18]. For instance, recent studies in a variety of chronic diseases found that patients experienced hardly any, or none at all, advantages of completing PROMs [19–21] In addition a Cochrane systematic review concluded that PROM completion makes no or little difference to patients regarding their perception of health and social functioning [18]. In contrast, positive results are also found, for instance in a quantitative study that reported a positive influence of PROMs on patients’ self-control [22]. In summary, there is ambiguous evidence that the use of PROMs helps to improve care processes from the patient´s perspective [8,23].

Our study focuses on the introduction of PROMs in the patient group with end-stage kidney disease (ESKD). The worldwide population of patients that need renal replacement therapy is estimated at over two million [2,3], while dialysis treatment incurs high healthcare costs and places a large burden on the health-related quality of life (HRQOL) of patients [24]. Also, although dialysis is seen as a high-tech treatment, there have been only a few major innovations over the last 50 years from the patients’ perspective [25]. In addition, symptoms and disease burden are not always recognized by clinicians, where nurses seem to be more accurate than nephrologists [26,27]. Because of the potential benefits of PROMs and indications that PROMs can be of added value to patients with chronic kidney disease (CKD) a standard PROM set was developed for dialysis patients in Dutch renal care [28,29]. We focus this study on the early implementation of this PROM set in Dutch dialysis centres, where early implementation is defined as the phase where ‘the decision to introduce the new dialysis PROM set in the dialysis centre has been made and professionals are actually working on implementation in the centre’.

We focus on early implementation because most studies on PROM implementation were conducted either before the implementation process started, in the context of a pilot study or after it was fully implemented [17,20,21,30–35]. These studies mainly focus on the development of a PROM set, the collection, administration and evaluation. Patients and clinicians are often involved in the studies. However, what is happening in a real-life setting during the early implementation phase is rarely studied and we argue that this may lead to a deepened understanding of the challenges that face the implementation of PROMs. Because of the importance of involving patients in designing the implementation process [36], we explicitly involve patients as participants together with clinicians.

The aim of this study is to understand the barriers and facilitators that both patients and professionals, as primary users of the dialysis PROM set, encounter during the early phase of its implementation and the challenges they face in realizing the claimed benefits of PROMs. Our findings can contribute to a better understanding of the operationalization of PROMs in daily practice, which may increase the likelihood of their sustainable use.

## Methods

We have performed a qualitative study using semi-structured interviews and reported the study in accordance with the Consolidated Criteria for Reporting Qualitative Research (COREQ). The study protocol of this research was approved by the medical ethical commission (METc) of the University Medical Centre Groningen in the Netherlands (METc number 2019/033).

### Setting

In October 2018, a dialysis PROM set was introduced in the Netherlands. Dialysis centres are encouraged to use the set, however every centre is independent on how, when and if they introduce PROMs in their centre. The set was developed in close cooperation with Nefrovisie, the Dutch quality institute for nephrology care, and the Dutch Association of Kidney Patients (NVN) and has been accepted as the standard set by all 95 dialysis centres in the Netherlands that together take care of 6,300 dialysis patients. The dialysis PROM set has a solid scientific base and its development is supported by a qualitative and quantitative study that is extensively described by Van der Willik et al [29,37]. The set consists of the SF12 health survey questions [38] plus the 30 questions forming the Dialysis Symptom Index (DSI) [39]. Before the nationwide introduction a pilot study was held in 2016–2017 involving 16 participating dialysis centres and, in addition, patients participated in focus groups. The pilot study showed a highly differentiated pattern among centres, with patient response rates to the PROMs questionnaire varying from 6% to 71%, with an average of 24%. The highest response rates were found in centres with high engagement of professionals. Nevertheless, the pilot study also illustrated that patients were generally positive about PROMs and appreciated the feedback given by their caregivers [37].

The introduction of the PROMs is supported on a national level by Nefrovisie and addresses many of the considerations as described in the User’s Guide to Implementing Patient-Reported Outcomes Assessment in Clinical Practice [40,41]. This central support offers dialysis centres with a variety of implementation strategies–like central IT support, newsletters, meetings, an informative film for patients, leaflets and factsheets on the website of Nefrovisie. A part of the site is dedicated to PROMs and how to start with PROM implementation in a dialysis center and a frequency of two PROM questionnaires a year is recommended.

**Fig 1:**
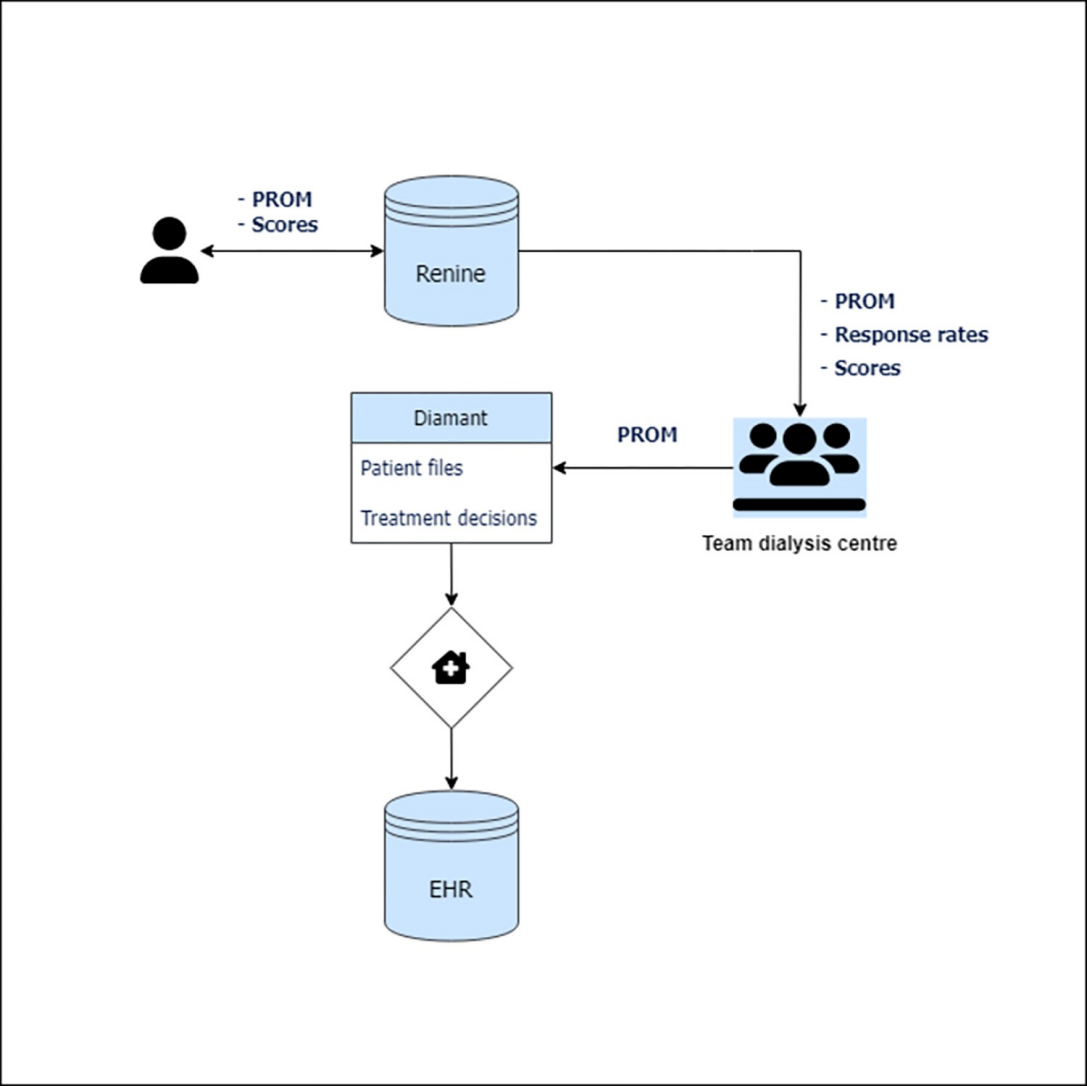
Involved IT systems.

A digital approach was chosen to minimize administrative burden, a known barrier in the implementation of PROMs [31]. Patients answer the PROM questionnaire online, get an immediate response with an overview comparing their scores with aggregated reference data and they can fill in their email address and read the results afterwards. The PROMs are stored centrally in ‘Renine’, the Dutch registry for renal replacement therapy to which dialysis centres have access. Who in the centre can access patient files is up to the dialysis centres to decide. In addition individual files can be downloaded into Diamant, a specialized system to store patient files and patient treatment decisions in the dialysis centre. How and if files are shared with the hospital’s electronic health records (EHR) is up to the hospital. See Fig 1 for a schematic overview.

The registry system Renine provides the centres with feedback on how many patients responded to the PROM questionnaire and the average DSI and SF-12 scores on the dialysis centre level.

### Data collection

We have performed a qualitative study using semi-structured interviews with both professionals–doctors and nursing staff–and patients. A qualitative method was chosen because we wanted to explore and understand the considerations and beliefs regarding facilitators and barriers of both patients and professionals during the early PROM implementation phase. Qualitative research is frequently used to understand beliefs, experiences and interactions of participants [42,43]. We developed a semi-structured interview protocol based on the Measurement Instrument for Determinants of Innovation (MIDI) [44]. Determinant frameworks, such as MIDI, specify determinants that influence implementation outcomes [45]. We considered the MIDI questionnaire framework as suitable for our aim because it addresses the domains of both users and end-users, in our case professionals and patients respectively. The interview protocol is presented here as S1 File (patients) and S2 File (professionals). We pilot-tested the interview questions with two professionals (one doctor, one nurse) and one patient. All three received a written summary of their interview and gave feedback. We concluded that the interview protocol met our information needs and that no adjustments to the protocol were required.

The selection of centres to be included in our study was made by Nefrovisie (MH) based on the criterion of being in the early implementation phase of PROMs and agreeing to participate in the research. Once permission had been granted, the researcher (WS) contacted centres by email or phone. Two of the centres had some earlier experience with PROMs having been participants during the pilot study. Patients were selected by a professional coordinator, either a doctor or nurse, within the dialysis centre. An inclusion criterion was that they had at least once filled in the dialysis PROM questionnaire. Interviewees were chosen on the basis that the selecting professional believed they might contribute to the research, would be willing to participate and, in some cases, were available for an interview during their dialysis hours. Appointments for the interviews were made by the centre or directly between the researcher and patients, based on convenience. The interviews were carried out from January 2019 until October 2020. All the participants signed informed consent forms. Especially when interviewing the patients, we could benefit from a mutual understanding and empathy because the interviewer was, as a former dialysis patient, familiar with the impact and routine of dialysis. As such, to a certain extent, WS can be viewed as an insider researcher by being part of the dialysis patient community [46]. We had an open conversation with patients, letting them set the pace and allowing them to raise topics that they saw as important regarding the potential use and expectations of PROMs.

### Data analysis

The transcribed individual interviews were analysed using open coding with the predefined MIDI determinants used as a starting point for the analysis. During the process of analysing the results, we decided to switch to an inductive way of understanding. We saw that the data we had collected were rich, and the use of inductive reasoning led to useful insights on both facilitators and barriers. As such, we consider the overarching themes of the barriers and facilitators that emerged to be a more accurate reflection of reality and we present the results accordingly, following the inductive themes and not the predefined MIDI determinants. The process of coding, then discussing themes, barriers and facilitators was iterative using the insights gained by the main researchers (WS, KA and MdJ). The afterwards calculated kappa value was above 0.81 which shows a substantial interrater reliability [47]. After analysing 17 interviews we reached a point of data saturation. The coding process was supported by the software of Atlas.ti, version 8.2.

To consider the trustworthiness of this study we refer to the elements credibility, dependability, transferability and confirmability [48]. To enhance the credibility of the data all authors were involved during the research from the study design to discussing the themes and interpret the results. To ensure dependability we described how we collected the data and how we analysed the transcriptions deploying an iterative process supported by coding software. With regard to transferability, although this study is focused on PROMs implementation in dialysis centres, the findings could well be used in other healthcare settings especially in chronic care management. Confirmability was assured by having critical discussions within the research team, continuous checking of concepts and exchanging information.

## Results

In total we held 23 interviews with 27 participants, with no one we asked refusing an interview. All interviews were recorded and transcribed verbatim. Three interviews with professionals were multi-person, one couple of two nephrologists and one couple of a nephrologist with a nurse. We also interviewed one group of three participants, of which two nurses and one secretary. The 13 professionals interviewed (doctors, nurses and secretary) were located in nine different Dutch dialysis centres across the Netherlands, four based in university hospitals and five in regional hospitals. During the interview only the respondent(s) and the interviewer were present. Six of the 13 professionals were nephrologists (3 female, 3 male), six dialysis nurses (all female) and one secretary (female). The average length of these nine interviews was 42 minutes, varying from 29 to 51 minutes.

Fourteen patients receiving dialysis treatment were interviewed individually, of which twelve were receiving in-centre haemodialysis and two peritoneal dialysis. The period patients received dialysis treatment varied from 2 years to 21 years, with an average of six years. Interviews were located either at the patient’s home (n = 5), during dialysis in the hospital (n = 5), in a private room in the hospital (n = 2), outside but close to the hospital (n = 1) and by phone (n = 1). The last two due to COVID limitations. During the interviews taking place during dialysis treatment, nurses would be walking around and other patients were also present. In three of the other cases, a family member was present in the background. In all these situations, no one intervened and the interview was strictly a one-to-one interaction. The average length of these fourteen interviews was 28 minutes, varying from 13 to 51 minutes. Three patients were female and 11 male, with ages ranging from 42 to 85. Two of the patients had completed the PROM questionnaire during the pilot study. We found that two of the patients we interviewed did not complete the PROM questionnaire and they were excluded from the analysis. Table 1. describes the main characteristics of the included patients.

**Table 1:** Patient characteristics.

Patient Nr.	Age (y)	Gender (m/f)	Years on dialysis	Dialysis modality	Location of interview
1	65–70	M	2.5	HD	Home
2	65–70	M	4.5	HD	Centre,
3	70–75	M	NA	HD home	Centre
4	55–60	M	2	HD	Centre
6	40–45	F	21	HD	Centre
7	80–85	M	7.5	HD	Phone
8	50–55	F	2	HD	Near centre
9	75–80	F	7	HD	Home
10	60–65	M	8	HD	Centre
11	40–45	M	15	HD	Centre
12	70–80	M	4	HD	Centre
14	50–55	M	20	PD	Home

HD = Haemodialysis.

PD = Peritoneal dialysis.

The analysis of the data resulted in four second-order themes of barriers in the early phase of PROM implementation in the care of dialysis patients: patient indifference to PROMs, scepticism on the benefits of aggregated PROM data, the limited treatment options open to doctors and, finally, organizational and operational issues. Apart from these barriers, we also identified several facilitators that help the implementation of the newly developed dialysis PROMs. These could be grouped into four second-order themes: professional involvement and patient support, a growing understanding of the use of PROMs, quick gains from using PROMs, and a clear ambition on patient care such as a shared view on patient involvement and management support. An overview of barriers and facilitators, together with illustrating quotes is presented in S4 File. In S4 we relate the MIDI determinants to corresponding barriers and facilitators.

### Barrier 1: Patient indifference to PROMs

We found that whether PROMs are considered valuable by a patient depends partly on the patient’s characteristics and the relationship developed between nurses and patients during dialysis treatment. In general, patients felt indifferent regarding the use of PROMs, not yet feeling that PROMs added to the quality of their treatment. Many could not recall completing the questionnaire although professionals had assured us that all had responded with the exception of two patients who were yet to be given a PROM questionnaire. Below, we highlight the main reasons for this apparent indifference.

#### Lack of urgency

We noted a lack of any sense of urgency by patients with regard to completing PROMs because they already interacted intensely with their nurse and doctor. Interviewees felt that a lot of information was already being shared since they interact with their physician during their weekly dialysis sessions. They visit their centre three times a week for four hours and while there share personal and social information with their nurses as is illustrated by: “The most senior dialysis nurse normally connects the patient once every one or two weeks to the dialysis machine. That’s the moment to have a deeper conversation with their patient.” (nurse 6). Patients confirm this as one patient explained: “I expect little from this [discussing PROM feedback; WS]. I filled in the questionnaire because they asked me to, but we have the opportunity to speak with the doctor or head nurse every Monday.” (patient 6). Another patient confirmed this: “After connecting you [to the dialysis machine; WS] on Monday morning they always first ask: ‘Do you have anything you want to ask the doctor?’” (patient 8). The same patient had been told not to wait for any questionnaire if they had questions for the doctor. Further, one patient indicated that they expected the nurse and doctor to communicate with each other: “I presume that the nurse also helps prepare the information for the consultation …” (patient 5).

A nurse who had access to PROM results confirmed that PROMs do not necessarily bring new information to light: “We know the patients so well, to us they [PROMs; WS] offer little new information but it does make the conversation a little easier.” (nurse 2). Patients reported themselves to be to be fairly content with their current patient–doctor communications, and PROM questionnaires were not, or only slightly, perceived to contribute to improved communication. Finally, it was noted that unless doctors provide some follow-up to the questionnaire during a consultation it will be even more difficult to motivate patients to respond to the PROMs in the future: “There has to be a follow up, because when the doctor doesn´t discuss it, it´s a waste of our time and that of the patient.” (nurse 2). One patient explicitly remembered discussing the results with a specialized nurse: “Later on, I had a conversation about it with the specialized nurse. I got compliments that I maintained such a positive attitude.” (patient 7).

#### Questionnaire fatigue

During the interviews, patients expressed hardly any real interest in an additional questionnaire. Both professionals and patients reported that questionnaires are part of everyday life for dialysis patients. A consequence of this was a low motivation to complete the PROM questionnaire: doctors and nurses had to put in serious efforts to motivate their patients to fill in the PROM questionnaire. Patient motivation is an issue for professionals as was well-illustrated by one doctor who stated: “The problem we have is that our patients are overwhelmed by all these questionnaires. They suffer from questionnaire fatigue. We really had to put some effort into this to motivate them to respond.” (doctor 3). However, most patients did not have strong objections to yet another questionnaire.

#### Patient characteristics and trust

The willingness to complete the questionnaire also depends on patient characteristics such as age and critical attitude towards their doctor as was illustrated by a nurse: “Young and more opinionated patients say ‘I don’t agree, I’m not going to do this’ … the older generation do as the doctor says, the younger generation asks ‘why should I do this, what’s in it for me and what can I do with this?’. So yes, that’s different.” (nurse 2). We found that the willingness to share personal information, which is a part of PROM questionnaires, is also linked to the trust relationship between caregiver and patient. Low trust might hamper open communication between doctor and patient, as was illustrated by a patient who said: “For a few weeks now, I have a new doctor. My early experiences don’t encourage me to be more open. I don’t know him yet and I’m not going to tell him everything.” (patient 5). Another patient was very explicit about the importance of trust: “I’m here for dialysis but I had surgery eight years ago in another hospital. I go there once a year and I always tell more there than here. It’s a matter of trust. … Over there I have already had the same doctor for eight years. Here, it changes every time.” (patient 9). A nurse added that PROMs might be particularly helpful for patients who are not open in their communication due to their personality: “In my opinion. we have a group of communicative patients and a group that are more closed. To them this [PROMs; WS] may offer an opening.” (nurse 2).

### Barrier 2: Scepticism on the benefits of aggregated PROM data

The second theme on barriers that we identified concerns clinicians’ and patients’ scepticism on the potential benefits of PROMs. We describe them in four subsections that each highlight a different aspect of potential benefits from a different perspective: the usefulness of comparing aggregated PROM data according to patients and clinicians, the feasibility of comparisons according to clinicians, the limited possibilities of aggregated PROMs to reveal differences in HRQOL as an indicator of quality of care and the difficulties with PROMs of following patients’ wellbeing over time.

#### Doubts: Are comparisons useful?

The aggregation of individual PROM data could help in comparing outcomes of different dialysis modalities—such as in-centre versus at-home dialysis—and to compare patient-reported outcomes between centres as an indicator of quality of care. To compare aggregated results needs enough data to be collected over time and a general acceptance of PROMs in the various centres. However, doctors are reluctant to accept the results of any comparisons, as a doctor explained “Whether my patients are doing better or worse than those in a hospital 100 kms from here? I don’t think that is relevant.” (doctor 2). One patient stated his feeling on this as follows: “I’m not interested in PROMS as quality indicators. I have my own personal support here and I feel at home here.” (patient 5). This illustrates the broader impression gained that patients are not very interested in outcome comparisons between dialysis centres. Although eager to improve healthcare practices, doctors did not consider aggregated PROMs as an adequate measure to compare the quality of medical care as was illustrated by one doctor stating: “I’m sure we will find some differences, however I don’t think this will be due to differences in the quality of care.” (doctor 4).

#### Doubts: Are comparisons feasible with large case-mix differences?

Doctors reported concerns on the feasibility of aggregation and comparison because of differences in the case-mix of patient groups. Professionals argued that case-mix differences are widespread across and within dialysis centres and goes beyond differences such as dialysis modality, age, causes of renal failure, social differences and occurrences of comorbidity. As one doctor emphasized: “I believe that if you make nationwide comparisons between centres, you should declare the academic hospitals as a special group and, even within them, there are differences.” (doctor 3).

Doctors, referring to the possible use of outcome differences by health insurers, stated: “Centres might develop a defensive attitude if that happens. Such as by only offering PROMs to their best patients.” (doctor 3) and “I think that would be dangerous. It’s so difficult to compare centres.” (doctor 6), another added “Our mortality rates are rather high.” (doctor 5). Improving care processes based on benchmarking, even though potentially attractive, was considered to be difficult if based solely on PROMs.

#### Doubts: Do aggregated PROMs reveal differences in HRQOL?

PROMs are often presented as a means to measure HRQOL, but doctors raised serious doubts regarding this during the interviews. PROMs might reveal something about the quality of care, as one doctor explained: “It is possible that, for instance, we’ll see differences in terms of a symptom such as cramp. That might say something about the quality of care.” (doctor 4). A nurse had a similar view: “Imagine we have low scores on the sexual functioning of our patients. In that case, we could ask ourselves ‘are we giving enough information?’ … ‘are we doing enough about it?’.” (nurse 4). However, there was a reluctance to use PROMs as an indicator of differences in the quality of life, and attributing this to better treatment in one centre than another: “Suppose we find a higher quality of life in medium-sized municipality A as compared to patients in a densely populated urban city environment B. So what? In B, there are many more patients with underlying social problems, such as lower incomes, a high percentage of immigrants and higher unemployment rates. These patients have more health issues. I don’t think you can blame healthcare for this.” (doctor 4). None of our interviewees expected any meaningful aggregated information from the PROM scores regarding HRQOL issues. This was illustrated by one doctor who argued: “The whole quality of life thing? We couldn’t even find differences between night and regular centre dialysis. And, in my experience, the first group tells me they feel better.” (doctor 4).

#### Doubts: The difficulties in following patients over time

Collected PROMs data can not only be used for aggregation and comparison purposes but also to provide time series information on individual patients. Similar to time series data on medical laboratory results, it could be valuable to follow patients over time and adjust medical and supportive decisions accordingly. However, in order to do this using PROMs, doctors need patients to fill in the questionnaires on a regular basis. Here, although Nefrovisie recommends a distribution of dialysis PROMs, we found that in practice PROMs were only distributed among in-centre haemodialysis patients once a year prior to their scheduled annual extended consultation with a doctor. This was because professionals felt that the yearly consultation was the right moment to discuss the PROMs with their patients, “We want to distribute the PROM prior to the extended consultation, which is once a year.” (doctor 5). This low frequency may hinder the effective monitoring of patients over time.

Further, the professionals interviewed emphasized that the conditions of patients undergoing dialysis may well deteriorate over time and that changes in PROM results may not represent the quality of care but, rather, that a greater burden of symptoms may simply reflect their changing medical condition. An interesting aspect related to time-series data is that several doctors mentioned that the symptom burden experienced by patients, as measured by PROMs, can apparently improve while their medical conditions are worsening. They explained this phenomenon by the improved capabilities of patients to accept and cope with their situation. As a nurse described: “Our patients’ physical condition may deteriorate severely over time and still their quality of life score remains on the same high level (nurse 2).

### Barrier 3: Limited treatment options open to doctors

As the third barrier theme, we saw that doctors did not always have adequate treatment options for the symptoms and poor health outcomes reported by patients. It was also reported that not every doctor is interested in taking a broader view on patient treatment.

#### Are dialysis doctors motivated?

Referring to the willingness of some colleagues, one doctor argued “Not every doctor invests in the annual extended consultation with their patient. … To put it bluntly, some nephrologists see dialysis treatment as a tick-box exercise.” (doctor 4). This doctor then explained that some colleagues do not consider dialysis as a very exciting and, for them, challenging form of treatment. PROMs are an addition to the doctor’s toolbox, and not all doctors are equally motivated to put in the extra effort required.

#### Are doctors able to adequately intervene?

Another challenge that arises is linked to doctor’s capabilities and core speciality. Some indicate they can perfectly well intervene on medical problems, but are not confident over what to do about other patient-reported complaints that go beyond their profession. This was clearly indicated by a doctor who said: “I think PROMs can be a problem for some doctors who will find it difficult to discuss complaints that they cannot do anything about. … Even to me this is a bit frustrating.” (doctor 3). Doctors could find it frustrating to ask people about complaints when they cannot offer any guidance, intervention or support. As another doctor mentioned: “A sexual condition can scare me off and I think ‘How can I help?’. The most common response is to refer to a urologist. … And the same with sombreness and depression.” (doctor 6). The tension between asking about symptoms and the perceived lack of treatment options, and the discomfort that then arises, was summarized as follows: “In my opinion, if we ask these things of patients, we also should offer them adequate follow up. I think we are still struggling with this.” (doctor 6).

#### Patients’ preferences and protocol conflict

Professionals also reported a potential conflict with existing medical protocols. PROMs intend to give the patient a voice and more saying in the way they are treated. However, patients’ preferences may be in conflict with existing medical protocols. For instance, if patients were to indicate that their HRQOL improves with fewer dialysis treatments they may then ask doctors to deviate from the standard protocol of three four-hour sessions a week, where from a medical standpoint such a reduction would amount to inferior treatment. This potential conflict was clearly expressed by a nurse: “We are assessed on achieving good lab results, but maybe the patient only wants to undergo dialysis twice a week and this improves his quality of life. In terms of the visitation review, we are doing a bad job–but the patient is happier. We have many patients who really do not want a shunt to dialyse, they prefer a jugular catheter. This places us in a bad situation regarding professional guidelines that prescribe the maximum percentage of patients with a jugular catheter.” (nurse 2). Here, it is important to note that compliance with protocols is safeguarded through external visitations. As a result, nursing staff who are in many cases responsible for distributing PROMs and motivate patients to respond, might be reluctant to do so if they feel that PROM results may not lead to changes in treatment and that patient preferences are disregarded due to protocol restrictions.

### Barrier 4: Organizational and operational issues

#### Procedural growing pains

Several centres started enthusiastically, quickly giving PROMs to all of their patients. However, they soon discovered that this procedure could be improved because the conditions of some patients could change in the considerable period that elapsed between answering the PROM questions and their scheduled annual consultation such that the PROM results were no longer adequate. In addition, some centres felt they had to think carefully about other PROM routing issues such as distributing them twice a year might consequently double the number of extended consultations to also twice a year. The PROM might also lead to changing the timing of the annual consultation to before or after the annual multidisciplinary team meeting where patients’ conditions are discussed within a team of various disciplines.

It was also discussed during the early implementations whether a nurse should also attend the yearly consultation with the doctor and the patient, because the implementations had revealed that nurses being present might also help improve communication. A nurse described how it could be logistically difficult to distribute PROMs twice a year: “We currently combine it with the annual consultation, and it cannot be right that, a second time, the PROM is not discussed with the patient.. . . That still puzzles us.. . . Maybe we just have to decide to keep it to once a year.” (nurse 2). All these possible adjustments to existing routines brought their own planning questions. Overall, the early implementation stage was seen by several of the professional respondents as a learning experience in how to deal with the procedures.

#### Not only the doctor, but the whole team is needed

Although PROMs are a tool to enhance communication between patient and doctor, interviewees described how, to make this possible, the whole dialysis centre team has to be involved in the implementation process. Both doctors and nurses indicated that the efforts necessary to get PROMs distributed and returned are mostly put in by nursing staff and the secretariat who, as recognized by the doctors, already have a high workload: “It doesn’t take much time from me, but the secretary staff and the nurses, yes it takes them extra time and, nowadays, their workload is already quite high.” (doctor 2). The dialysis team as a whole is reported to be crucial to PROM implementation and it was also described how a larger team needs more effort in terms of coordination and motivation to handle the implementation. “All personnel have to be involved if you are striving for a good end result. Knowing my department, I think we will manage this. We only have a small team, which is convenient.” (doctor 1). Furthermore, as the doctor explained, the demands on a centre’s team can be more complex due to factors such as having multiple locations and a high employee turnover: “A big dialysis centre with more locations, well … then you would need a more structured approach and you have to train all those teams.” (doctor 1).

#### Nursing staff: Pain but no gain

Although the importance of nursing staff when working with PROMs was widely recognized by the doctors, several nurses still reported that the information generated by PROMs is often only seen by doctors and patients, with nursing staff being excluded. Some saw this as somewhat unjust as nurses are very close to the patients and spend up to 12 hours a week with them, much longer than the doctors. Further, the non-coded information that nurses gather during dialysis treatment could be valuable but is not normally considered during the annual consultation. Nurses know a lot about individual patients, but this information is not systematically used, as a nurse explains: “I have never noticed that doctors asked us as nurses how we see things. It happens in the multidisciplinary meeting, but by then the annual consultation has already taken place.” (nurse 6). Although this does not directly affect PROM implementation, a feeling of being excluded is not very motivating.

#### Interference from external and internal turmoil

In terms of organizational barriers, the professionals interviewed described that mergers, rapid changes in personnel, organizational turmoil, an overload of projects and organizational changes can all interfere with PROM implementation. The timing of the implementation project is therefore important and can be disrupted by external or internal turmoil. One doctor explained this as follows: “I have to motivate colleagues to work with this [PROMs; WS] so I must create acceptance and, even when people change jobs and functions during changing alliances, we have to continue our way of working.. . . Especially in these times with a lot of turmoil [a relocation of the department; WS] I believe that good projects can die because they are started at the wrong moment. To me this is a concern.” (doctor 5).

#### Inevitable IT nonalignment

Finally, as part of the operational barriers, interviewees reported that workflow difficulties were arising from the fact that the centre’s dialysis patients’ medical records and the PROM results were kept in a dedicated IT system called Diamant. Interviewees indicated that although results were available in Diamant they could not be accessed through the electronic health record (EHR) system used by the wider hospital. This was an issue because, during the annual consultation, doctors were required to use the EHR system, and not Diamant. As a consequence, these two systems were being used in parallel, increasing the workload when working with PROM responses. A nurse stressed the need for information integration: “We are now printing the digital [PROM; WS] file and then scanning it for insertion into the EHR.” (nurse 2). This was supported by a doctor stating: “A connection with our EHR system would be great” and “At the moment it brings additional paperwork, if I want PROM results in the EHR I have to retype them so to speak.” (doctor 2). To address this problem, sometimes PROM data are printed out and delivered to the doctor manually. For the short-term, doctors see this as somewhat acceptable, but in the long-term they indicate that this cannot continue because it is too complex and time consuming for everyday routines.

### Facilitator 1: Professional involvement and patient support

#### Involving professionals as implementers

Once the decision had been made to use PROMs, we found that organizing the PROM implementation could be done by a project team with a few interested colleagues, quality assurance nurses or secretariat staff in cooperation with a nephrologist. Having preparations made by one or two coordinators, rather than doctors, can facilitate the daily use of PROMs in practice. The interviews revealed that the coordinator could for instance be a nurse who already has responsibilities in the field of quality assurance or is following a career path where the coordination of PROM implementation is part of a study trajectory. As one nurse described: “It was decided [PROM implementation; WS]. We just had to implement it. Because I am studying to become a quality assurance nurse I thought it would be perfect to choose this as a topic for my thesis.” (nurse 6). During some of the interviews, it was described how the coordinator, a healthcare professional, managed all the preparatory operations such as the distribution of PROMs, staff training and motivation in cooperation with the centre’s management team. The planning and distribution of PROMs support was arranged by a central point in the various dialysis centres, for instance by a secretary or support staff such as a quality manager or nurse.

#### Best practices and lessons from previous experience

Respondents argued that implementation was also facilitated by having insights into ‘best practices’ and learning about implementation and daily use of PROMs from other centres. As one nurse explained: “Of course I use the Nefrovisie website and I read a lot there. When I’m sitting at home and I’m scrolling then I think, ‘yeah, that’s also a good idea’. … This is very convenient and useful to me. I like it.” (nurse 6). Gathering information was reported as being done directly, by mail or phone, or by dedicated newsletters from Nefrovisie. Earlier experiences with PROMs, for instance in a pilot setting, also helped to develop the appropriate operational procedures. Such experience was argued to be a motivation because it showed that the time and effort necessary to implement PROMs were worthwhile. One centre had already been using a similar questionnaire and the switch to this PROM set was not perceived to be a major change. This was explained by a nurse when explaining the switch from their original questionnaire to PROMs: “Yes, that helps of course, because it already felt familiar. The procedure was already known, so in fact not much has changed.” (nurse 4).

One doctor, talking about earlier experiences during a pilot study with the PROMs, said: “When discussing the pilot PROM results with patients I found it very informative that patients have more complaints below the surface than those I discovered during regular visits.” (doctor 1). A very practical lesson that could be learnt from other centres is that a high response rate is not easy to achieve without hard work by the whole team. A doctor explained: “We had a very high response rate, but you really have to make an effort for this. These [dialysis patients; WS] are patients who usually are very tired, make many visits to the hospital and we already ask a lot of them. So, if they don’t see the benefits then it is very hard to get a response.” (doctor 2). The key to achieving a high response rate from patients, as argued by the doctor, was persistency in urging patients to return the PROMs. Another potential lesson is that PROMs are a practical tool that is not difficult to use. Here, several coordinators, nephrologists and patients reported that the questionnaire is easy to understand and easy to complete, with neither patients nor doctors reporting high barriers that had to be overcome to interpret the results.

#### Organizing support for patients with low health literacy skills

A doctor explained that some patients may have difficulties with the questionnaire, and require special attention and support: “In particular, the patient who finds this difficult is the patient that also has difficulties expressing himself to the doctor. … It often seems that this type of patient says they are feeling okay but, in reality, there is much more to it.” (doctor 2). It was also argued that it was particularly reluctant or poor literacy patients that needed to be involved because they were the most likely to surprise their doctor with new information they had not shared before.

Respondents described several preparatory activities that could achieve a higher response rate. These involved preparing to help and support patients in completing the questionnaires, linking the annual anamnesis assessment that has some of the same questions with PROMs, providing material support such as tablets, and giving patients the freedom of choice as to where and when they respond, either at home or during dialysis. A patient commented on this: “At home I feel more at ease. (..) I do know how to use a computer but I’m not a freak. Settings are always a bit different and I’m more comfortable when I use my own computer.”(patient 3). With regard to the PROM questionnaire itself, some respondents indicated that PROMs are a practical tool and in practice could replace existing, more complex, questionnaires. This was well illustrated by one coordinator who stated: “We were looking for a method to be more patient-oriented. … We started looking at the positive health perspective and we were almost ready to introduce this but, then, PROMs came along–a beautiful solution specifically targeted at our patient group.” (nurse 1).

### Facilitator 2: A growing understanding of the use of PROMs

#### Learning to assess patient responses

Professionals indicated that increasing experience with using PROMs helps them in understanding patients’ responses, intervention options, possibilities for in-centre comparisons and differences in symptoms between centres. One doctor explained this as follows: “I think that’s a matter of experience. An item score of 15 or 30 means nothing to me but, at some moment, when you use questionnaires more often, then you master it yourself.” (doctor 6). These possibilities may develop over time as more centres and more patients participate provided there is an adequate learning community among professionals and an active exchange of ideas, experience and knowledge. One patient also described this learning curve, illustrating that patients may also need some time to learn: “At first we were thinking ‘what’s this, all these questions?’. And then we filled them in, and we checked the scores. And well, it is a good thing to do this once.” (patient 7). However, the same patient was not enthusiastic about the idea of a repeated questionnaire: “If they would ask me again, I would say: no, rather not.” (patient 7).

#### The power of using one standard PROM set in dialysis

It was argued that a necessary condition of learning as a professional group is to use the same PROM set across all related centres. As a doctor explained: “It would be nicer if we all used the same questionnaire in haemodialysis and other CKD treatments, used it in the same way and built experience in the same way. … At the moment, similar things are worked with, in many isolated situations. That is not necessarily wrong but, if we really want to do something with it in the Dutch nephrology scene, then joint actions would really be better.” (doctor 6). The acceptance of the new standard dialysis set thus enhances the use of a single PROM set and discourages the development of ‘local PROMs’.

#### Openness to share PROM experiences

Although the use of a standard set enhances the possibility of exchanging experiences, the willingness and openness of doctors to share their experiences through the use of PROMs is also a necessity as was argued by a doctor: “We have to share these experiences in national task groups and at congresses.” (doctor 5). Patients also appeared willing to share information, not for themselves but for improving care for other patients. Participating in research, such as in the study at hand, may give patients a sense of contributing to the healthcare community. One patient in explaining their motivation for completing questionnaires stated: “I always say, I’m learning from it, but also for another.” (patient 3). Another, to us unexpected, benefit from PROMs is that one patient discussed the PROM results with a family member. In this instance, the PROM results helped the patient share the information that was presented with family, and this helped to reach a better understanding: “What I liked was that afterwards [on returning the PROM; WS] I could print the results. … I mean, you are always talking about these things but now it’s on paper. … And my sister told me, what you have filled in and what your scores are, that’s exactly right.” (patient 7).

### Facilitator 3: Quick gains from using PROMs

#### Easy-to-use product

Related to the PROM set product itself, our interviews suggested that the introduced dialysis PROM set, co-designed with patient involvement and doctors, was ready to go: that it was easy to use, without extensive paperwork due to the digital approach, not too many questions but comprehensive and balanced. Both patients and doctors considered the PROM set to be very practical despite the efforts that still have to be made to actually put them into practice. One patient said “I think the list was extensive, but it wasn’t difficult” (patient 12). In addition on completeness and ease of use of the PROM set: “I think they are very complete. (..) And also I feel they are easy to fill in.” (nurse 2)

#### Receiving instant feedback

After completing the questionnaire and hitting the Submit button, results are reported back to patients in a matter of seconds, including their overall benchmark in relation to other patients and the wider Dutch population. The professionals in our study said that patients had indicated that this motivates them to respond, and doctors and nurses see this as a positive attribute. As a nurse described, patients appreciate this instant feedback on their questionnaire: “The beauty is, also for the patient, that they get their report straight away, all in colours, which is very convenient.” (nurse 3). The value of instant feedback was also described by a patient who had very thoroughly filled in the questionnaire and studied the outcomes. This patient paid attention to individual scores on the item list and to the average at the end of the questionnaire. The patient was not surprised by the results, but appreciated the confirmation given by the PROM results: “I found it useful. I already knew I’m physically not in good shape. … I mean, you always talk about it, and now it’s crystal clear on paper.” (patient 7).

#### PROMs as a practical tool and time saver

Doctors indicated that they regarded PROMs as a practical tool to support their consultation with patients. It helps them to gain a more structured insight into patients’ troubles, complaints and symptoms, and that this was a motivator to implement PROMs. Doctors informed that using PROMs helps them to broaden the consultation with regard to topics discussed. PROMs are also a time saver: they help doctors focus more effectively on the problems that patients themselves report. One doctor said about working with PROMs: “I believe them to be a beautiful tool to use during a consultation. … I see immediately the topics that I don’t need to ask about anymore. As in ‘are you having complaints about …?’ … I can immediately focus on the complaints that patients have reported, which is a great opening for further discussion. It doesn’t cost any time and I can directly aim to talk about relevant complaints.” (doctor 2).

Results are used alongside lab results on phosphate, haemoglobin, parathyroid hormone levels etc. The balance may vary with age as one doctor indicated: “These [lab results; WS] are especially important for patients with a reasonable life expectancy but, obviously, we treat many elderly patients with a lower life expectancy and especially to them the quality of life is very important.. . . A phosphate level of 2.0 or 1.7 doesn’t make much difference. Here, it is more important how this patient experiences his wellbeing. … And for me, what can I do to improve his life for the time he has left?” (doctor 2). Here, this doctor was demonstrating how PROMs are an aid to discuss issues that go beyond lab results for a specific patient group.

#### Easy handling and better consultations

PROMs are considered to be complete and are balanced to the extent that they are easy to work with and ask the right questions. Some questions might be added, but it is recognized that this would complicate the questionnaire. Doctors expect the quality of their annual consultations with in-centre dialysis patients to improve because of the use of PROMs. They also indicate that it especially helps when patients become actively involved and adopt an active communicative attitude. Operational fluency is also enhanced by the easy connection between Nefrovisie-Renine and Diamant. As a secretary put it: “I just have to press ‘save as’ to save the PROM pdf file from Nefrovisie-Renine in Diamant. … A matter of only a few small steps.” (secretary 1).

### Facilitator 4: A clear ambition on patient care

#### A shared view on patient involvement

A positive contextual element, related to the work environment of doctors, is having a shared view on patient involvement, both within the professional group of nephrologists and within a centre’s nursing team. This is not only regarding the PROMs, which can be considered to be a tool, but also regarding what the PROM set represents: another way of treating patients–with active patient involvement, shared decision making and more attention to non-medical issues. A nurse gave the following argument: “It’s different. It’s another way of how we work, another way of gathering information and getting different information. Not just medical, but also psychosocial. Yes, I believe this is very important.” (nurse 6). A doctor confirmed: “This is of course a topical issue nationwide, the whole issue that patients should have more say in their treatment. In many professional groups, you notice that PROMs are growing in importance. In my opinion this was not yet the case five years ago.” (doctor 2). Just like the doctors, the nursing staff also have an open attitude to embracing active patient involvement and this enhances the implementation of PROMs.

#### Clear leadership

Further, the interviews indicated that clear leadership has a positive effect. Here, firm statements at the beginning of the implementation process like ‘we are going to do this’ helps staff to accept PROMs and stresses the fact that PROMS are considered essential for the centre’s dialysis care. As a coordinating nurse stated: “There was no choice whether personnel would accept it or not. So, we are just going to do this. Some people see the benefits, others don’t and consider it to be nonsense. Well, come on. It just has to be done.” (nurse 2). This firmness may even be transmitted to patients: doctors might say ‘this is important, so I strongly advise you to fill in this new questionnaire, it helps with your treatment’. One doctor was very firm on this: “Some [patients; WS] did not respond and then we said: ‘Here is an iPad and you are just going to fill in the questionnaire’, actually being very directive, and ‘I am going to tell you the results.’.” (doctor 3).

#### Management support

Interviewees mentioned that acquiring the extra capacity to organize the introduction of PROMs, set up procedures, motivate colleagues and facilitate patients and staff takes time. Management support for this helps in succeeding. This support came from the management of the centre and not from the top management in the hospital. Several interviewees indicated that top management played no role in the implementation of PROMs, such as a nurse who stated: “We [dialysis centre; WS] are an island within the hospital. We do something because we think it is important.” (nurse 4). This finding indicates that dialysis centres are rather autonomous when it comes to decision-making.

## Discussion

To the best of our knowledge, this is the first qualitative study into the barriers and facilitators that arise during the early implementation of PROMs that considers multiple dialysis centres that are all implementing the same PROM set and includes the perspectives of patients, doctors and nurses.

The aim of this study was to understand the barriers and facilitators from a user’s perspective—both patients and professionals—during the early implementation of a newly developed dialysis PROM set. We found implementation to be a knotty process where users–patients, doctors and nurses–each assess PROMs from their own perspectives and with different expectations. These may either facilitate or hamper the use of PROMs.

### Patients

Although considerable attention is given to the involvement of patients in the development of PROMs [29,49], little consideration is given to the willingness and motivation of patients to complete PROM questionnaires. The focus has been on the development of easy-to-use questionnaires that will place only a small burden on patients. Nevertheless, when it comes to the dialysis PROMs, our data suggest that patients maintain a neutral attitude towards the use of PROMs and feel no extra motivation to complete them compared to more common questionnaires they are asked to fill in, for instance about the quality of care.

We identified this lack of patient motivation from the indifference that patients demonstrated towards the dialysis PROM set, and concluded that this was partly because they already communicate a lot with nurses during their dialysis treatment. Illustrating this was the fact that quite a few patients, although it was confirmed by the centre that the PROM had been discussed with them, did not recall discussing PROM results with their doctor and some even reported having no need for any discussion. This finding seems to go against recent PROM literature that suggests that patients value discussing PROM results with their doctor and expect there to be benefits [37,50]. However, Damman et al., when assessing PROMs related to Parkinson’s disease [51], also found mixed results on the felt needs of patients to discuss PROM data, and especially HRQOL questions, with their doctor, mainly because patients were too busy running their lives. It can be argued that our data suggest that patients perceive the PROM questionnaire as just another tool for doctors to employ. This could be due to the fact that, at the time the interviews took place, this PROM was not yet a standard routine in the dialysis centres.

Recent studies that included patients who at least responded twice to digitally available PROM questionnaires in the field of hematologic care and renal care had similar results on patient motivation, where patients did not consider PROMs as ‘a tool for them’ [19,21]. We argue that it may take some time for both patients and doctors to learn how to use PROMs in their communication and create additional value for patients. Or, as Staniszweska et al. state, using PROMs and creating patient benefits is a matter of evolution, not revolution [52]. The initial phase of PROM use should perhaps be seen as finding the right way, with adjusting procedures, to discuss the results during a consultation and to deliver support to patients that they recognize to be an outcome of the PROM questionnaire.

In the context of a dialysis centre where there is already close communication between patients and nursing staff in a long-term intensive care setting, patients’ motivation may well be initially low for what they themselves call ‘yet another questionnaire’ when the stimulus to respond does not seem to go beyond ‘following doctor’s orders’. We note that according to the Dutch dialysis quality protocol patient satisfaction surveys and regular intake questionnaires like the yearly anamnesis are also performed. In addition, Consumer Quality (CQ) scores, short-form Picker experience questionnaire and Edmonton Frailty Scale (EFS) exist for dialysis patients. Patient experience measures are encouraged by the Dutch Ministry of Health, Welfare and Sports [53]. All this can lead to questionnaire fatigue, or loss of interest and motivation.

Here, our results suggest that healthcare professionals, rather than patients, are the key driver in implementing PROMs and that some perseverance is needed during the early stages of implementation. However, for long term success, patients need to be enthused and their experiences and needs should be taken into account by health providers.

A claimed barrier to digital PROMs, such as the dialysis PROM set, is that some patients lack digital skills [1,54]. However, in this regard, the patients that we interviewed showed no hesitation and reported that the digital questionnaire was rather easy, a benefit of a web-based application that was reported earlier [55]. This digital acceptance could possibly be explained by the fact that the elderly, and the vast majority of patients on dialysis are over 65, have now generally developed digital skills. In addition, the new PROM set was developed with high involvement of patients and this may well have resulted in an easy-to-use questionnaire. Further, in some cases, if they considered it necessary, patients received support within the centre, where they could complete the questionnaire, or at home from their family.

Furthermore, we see that PROMs could be used as a tool to support a patient’s self-management actions in their daily life [56]. PROMs could make symptoms and concerns more visible and transparent to family, friends, employers and colleagues, and therefore not only help communication with doctors but also within the patient’s social environment. If patients were to receive some awareness training on the potential PROM benefits this could increase response rates.

### Doctors

We noticed that some doctors can feel uncomfortable about asking the same questions over and over again in different forms when there is a lack of appropriate interventions they can offer in support. This is in line with previous research that shows ambiguity over the benefits of PROMs in haemodialysis care and potentially may lead to a decrease in providers’ confidence in symptom management after using PROMs due to a perceived lack of intervention options that even left some providers with ‘a feeling of failure’ [33]. That doctors may have concerns about raising issues reported in PROMs that they feel they cannot deal with, was also reported in a study in oncology care [57]. We suggest that PROMs may well be effective in terms of boosting symptom awareness, but are not necessarily effective when it comes to symptom management. This disconnection between symptom awareness and symptom management was already being signalled in the early phase of implementation. To improve symptom management based on symptom awareness, and therefore see more benefit from the use of PROMs, professionals could increase their use of existing possible intervention options as suggested in earlier publications [58,59].

The use of aggregated PROM data as a benchmark for quality of care was questioned by doctors due to the large case-mix differences between centres. Recent research in a dialysis setting indeed suggests that case-mix adjustments have to be made [60], as has also been illustrated in stroke care [61]. In addition, doctors indicate that it is difficult, if not impossible, to interpret aggregated PROM results without knowing the patient, their comorbidity, their history and their personality. This is especially true for the SF12 questions that assess HRQOL elements. For example, a patient who has been on dialysis for several years, and has already adjusted their way of life, may, despite a lower health status, report a higher HRQOL than a patient who is recently diagnosed with a severe kidney disease, still has a relatively high health status, but worries about the impact on their life and the possible loss of employment. This phenomenon of patients’ shifting HRQOL expectations over time, also known as response shift [62–64], makes it very difficult, or at least challenging, to turn collected raw PROM data into meaningful medical quality-of-care information. Nevertheless, despite the doubts expressed concerning the feasibility of case-mix adjustments, some doctors emphasized that improvements in symptom management could be achieved provided that information on symptom management is openly shared and discussed between centres and among professionals.

In contrast with the largely neutral attitude towards PROMs expressed by patients, we observed that doctors showed a positive attitude in terms of the potential of PROMs to positively influence doctor–patient communication, as is also suggested in the literature [33,50]. It seemed that the majority of the doctors adopted PROM as a practical tool for one main reason: to improve communication with their patients. Barriers reported in the literature, such as difficulty in interpreting PROM results, scepticism on the use of PROMs in the consulting room and the time burden [4,65,66], did not seem to be experienced by our respondents. This difference may be due to the specific setting of dialysis treatment, where doctors and patients see each other frequently, in the case of in-centre dialysis even on a weekly basis, over many years. Therefore, the time burden is not an issue, and interpreting PROM results may be easier because doctors and patients already know each other. We found no scepticism by doctors on the use of PROMs in the consulting room. Instead doctors were curious about the impact of PROMs during consultations and were hoping that discussion of PROMs would lead to a better understanding about patients´ health related concerns.

The use of the PROMs was even mentioned as a time saver for doctors, a view corresponding with earlier research that indicates that PROMs allow doctors ‘to be a doctor again’ [5]. This is understandable because the PROM results make it easier for doctors to identify which issues to address with patients, rather than needing to run through an extensive list of possible symptoms as was usually the case during the annual consultation. It was also mentioned that doctors did not themselves spend much time on distributing and collecting the PROM questionnaires, that this was done by nursing staff, so the time burden for doctors outside the consulting room is low.

We argue that experiences from the early phase of PROM implementation can be used as a learning tool. Sharing early experiences, questions that arise and solutions found, could lead to a steeper learning curve on how to employ PROMs and take dialysis care to a higher level. All Dutch dialysis care centres now can use the same web-based PROM set with low administrative burden that may lower professionals’ barriers [55]. Although the implementation is organized differently in each centre, the group of professionals shared the view that PROMs could help in the communication between doctors and patients, however whether this turns out to be significant remains to be seen. Nevertheless, we see this shared ambition as a positive element in moving forward with the use of PROMs in dialysis care that is encouraged by supporting implementation strategies like training, meetings, a central helpdesk, informative newsletters and an up to date website full with tips and ideas.

Regarding integration of patient data, literature emphasizes the importance to integrate PROM data with the patient’s electronic health record to overcome practical barriers and to enable comparative effectiveness [67]. In our study we found that a hybrid model was chosen, with an external platform for data collection [68]. Patients and caregivers can access PROM results and comparative data collection is possible for dialysis centres and healthcare professionals, especially nephrologists. For (secretary) staff it is easy to import PROM results from the external platform to the Diamant system. Although this is solution, for now, technically workable, for further and sustainable use of PROMs a few puzzles still remain. In the consultation room doctors often use the hospital’s EHR system, that is not integrated with the dedicated dialysis Diamant system. Also, we found no literature on EHR integration that recognizes the use and importance of intermediate workflow systems and patient record systems as Diamant. In addition, independent of technical infrastructure, physicians still have to make a huge effort to correct for case-mix differences and to really understand the meaning of aggregated PROM data statistics [60,64].

### Nursing staff

Little attention is given in the literature to the crucial role of nursing and administrative staff when implementing PROMs. Although training and motivating staff and the administrative burden are mentioned [1,69], the interaction between patients and nursing staff and the role that nurses have in implementing PROMs are rarely addressed. However a recent study in cancer care described the experiences of nurses with PROMs and how they were ‘wishing for a strategy” to use PROMs in daily practice [70]. We argue that in-centre dialysis patients, who are undergoing treatment over many years three times a week for four hours each time, have a special relationship with nursing staff. This relationship in a haemodialysis setting has recently been studied [71] and it was found that patients rated nurses’ caring attitude even higher than nurses rated themselves on six out of the ten dimensions of Watson´s Theory of Human Caring [72] with high scores for humanity, hope, helping relationship, teaching, environment and needs. We posit that, in a dialysis centre, the interactions between patients and nursing staff are more intensive than the relatively short contacts between patients and their doctors. The importance of the tacit knowledge acquired by nursing staff was similarly recognized in an implementation study of PROMs in oncology care [73]. Recent research by Delmas et al. confirms that nurses are crucial in haemodialysis because, on many dimensions, they have the most intense professional relationships with the patients [71]. Further, Raj et al. [27] suggest that doctors should incorporate the knowledge gained by dialysis nurses because nurses recognize symptoms better than doctors.

Compared to doctors, we would argue that nurses have different, more-personal patient conversations not only on how patients are doing but also on what their needs are, what is worrying them and how they are participating in their social environment. This may well influence patients’ attitudes towards PROMs. In terms of implementing PROMs, nurses could have a more explicit motivating role towards patients to improve response rates, they could combine the yearly anamnesis with the PROM questionnaire, they could discuss the results of an in-between PROM with the patient to give it a greater sense of meaning and thereby facilitate a frequency of twice a year, and they could also intervene by referring the patient to, for instance, a psychologist or a social worker. All this could be done under the supervision of a nephrologist.

### Lessons for practice

To improve care from a patient´s perspective, long-term collecting of patient outcomes is necessary. We argue that our study shows that significant progress has been made to overcome practical barriers of PROMs, although some of them are still present. This has resulted in an easy-to-use PROM questionnaire for both patients and clinicians, a well-organised digital approach of collecting and distributing PROMs and a strong motivation of clinicians involved in the implementation. However, this not enough to get acknowledgement of patients. Today, patients do not recognize direct benefits of PROMs and to fill in the questionnaire they are mainly motivated by the argument that they are helping their doctor. In our opinion, the most crucial step forward now is to improve support interventions based on PROM results that patients report. When PROMs are used to improve patients’ lives, and this is clear to patients, than we feel confident that patient’s acknowledgement for the use of PROMs will follow. In summary, we suggest the next step is to close the gap between symptom awareness and symptom management.

### Strengths and limitations

Our study has some limitations. First, we only selected centres that were early adaptors of PROMs, which may result in a selection bias towards those who are more reluctant in the acceptance of PROMs. Some centres could not participate in this study because of COVID restrictions and had to postpone the implementation process. However, we believe it is reasonable to assume that this PROM set is widely supported within the professional group of nephrologists. Second, as a result of our choice to select centres that were starting to implement PROMs, we were confronted with a knowledge gap regarding potential PROM benefits between the professionals and the patients that we interviewed. The doctors were well informed on the subject of PROMs through meetings, publications and discussions whereas the patients had only been briefly introduced to the concept. With this knowledge gap and the low awareness of PROMs during the early phase of implementation, a number of the patients even found it difficult to remember completing this particular questionnaire. This knowledge gap is a possible limitation of the research, but we argue it is also as a positive outcome in that this difference in itself is part of the implementation process.

We consider it as a strength that we interviewed a diverse group of nurses and doctors from academic and non-academic centres, and also a diverse group of patients in terms of age, gender and duration of their dialysis treatment. These diverse interviews helped to understand what was happening in the daily setting of PROM implementation. Another strength of this study is that we managed to deepen understanding of patients’ considerations on the role of PROMs. Furthermore, another strength is that the results of the inductive coding process were discussed among the authors several times to prevent confirmation bias. Results of the analysis were discussed among all the authors, of which two are experienced nephrologists and one an experienced qualitative researcher in renal care. As a consequence of this continuous reflection on the results, we have no serious concerns over the internal validity of our findings. Also the interview protocol that we used was based on the proven MIDI questionnaire framework on implementation.

### Conclusions and suggestions for further research

The dialysis PROM set that we have studied was introduced at the end of 2018 in Dutch dialysis care and is gradually being implemented in multiple centres. At the time of the interviews, PROMs had not yet become part of the daily routine within the centres. Patients completed PROM questionnaires because their doctor asked them to, and were yet to feel that PROMs contributed to better communication with their doctor or a better sense of wellbeing. The main reasons for implementing dialysis PROMs is for doctors to gain a better understanding of their patients and the expectation that it will improve symptom recognition as reported by patients. However, the doctors perceived a lack of intervention options, and this could endanger the long-term use of PROMs. Further, physicians were unconvinced about the possibilities of aggregation and comparisons between centres and treatment results, mainly because of considerable differences in case-mixes. Finally, we show that nurses play an important role in the patient–caregiver relationship and that this relationship could further enable the implementation of PROMs.

To increase understanding of the implementation challenges facing PROMs, we would suggest further research on the motivation of patients to regularly complete PROM questionnaires over a long period of time. We would also suggest evaluating the assumed learning curve: whether it really takes place and whether it helps to meet doctors’ expectations on potential PROM benefits. In addition, we would encourage further exploration of the possibilities for improving patient care through supportive interventions, either by doctors or other relevant professional caregivers. Finally, the role of nurses in dialysis centres could be studied further to better understand and enhance the use of their tacit knowledge regarding their patients.

## Supporting information

S1 File Questions and protocol for patients. (DOCX)

S2 File Questions and protocol for professionals. (DOCX)

S3 File COREQ checklist. (DOCX)

S4 File Illustrating quotes and corresponding MIDI-determinants. (DOCX)
