# Supplementary material for: Facing the challenges of PROM implementation in Dutch dialysis care: Patients’ and professionals’ perspectives
Source: PLoS One. 2023 May 15;18(5):e0285822. doi: 10.1371/journal.pone.0285822 (PMC10184911; doi:10.1371/journal.pone.0285822)
Supplement: S1 File — (DOCX) [file pone.0285822.s001.docx]

**S1 Questions and protocol for patients**

All interviews were tape recorded and transcribed. An informed consent form signed by the respondents was required. Forms were administered by the researcher. Interviewees were informed about the aim of the research, and interviewees could decide to withdraw from the research at any time. Published results are anonymized.

1. Questions associated with the user (MIDI determinants 8, 9, 10, 11, 12, 16, 17, 18)

- To what extent does using PROMs have personal benefits/drawbacks for you?

- Could you describe the possible important goals for you as a patient? What do you expect from the PROM questionnaires? Which goals do you think will actually be achieved?

- What do you feel about your responsibility to answer the questions put in the PROMs? What motivates you to answer the questionnaire?

- In your opinion, how do you experience communication with your doctor? To what extent do you expect improvements through the use of a questionnaire like PROM? Can you explain?

- Why would you cooperate (or not) when you are offered the possibility of filling out PROM questionnaires?

- What motivates you to participate?

- How do you interpret the results of PROMs and how would you discuss the results with your doctor?

- How were you informed about the use PROMs? Which caregiver approached you (e.g., doctor, nurse)?

General question: what other comments do you have?
