# Supplementary material for: Facing the challenges of PROM implementation in Dutch dialysis care: Patients’ and professionals’ perspectives
Source: PLoS One. 2023 May 15;18(5):e0285822. doi: 10.1371/journal.pone.0285822 (PMC10184911; doi:10.1371/journal.pone.0285822)
Supplement: S2 File — (DOCX) [file pone.0285822.s002.docx]

**S2** **Questions and protocol for professionals**
All interviews were tape recorded and transcribed. A signed informed consent form from the respondents was required. Forms were administered by the researcher. Interviewees were informed about the aim of the research and could decide to withdraw from the research at any time. Published results are anonymized.

1. PROM product-related questions (MIDI determinants 1, 4, 5, 7)

- To what extent is it clear how you can work in practice with PROMs? Can you describe this?

- What is your opinion about the overall complexity of working with PROMs?

- How well do you think PROMs fit into your day-to-day consultation? Please clarify.

- In your opinion, does the use of PROMs offer important additional value to your patients? Can you explain how?

2. Questions associated with the user (MIDI determinants 8, 9, 10, 11, 12, 16, 17, 18)

- What personal benefits/drawbacks does using PROMs have for you?

- Could you describe the possibly important goals for your patients as you see it?

- What do you feel about your responsibility as a professional to implement and use PROMs?

- In your opinion, will patients be generally satisfied or better serviced if you use and discuss PROMs? Can you explain why? (possibly key question)

- Why might your patients cooperate (or not) when you offer them the possibility of discussing PROMs?

- How can you motivate your patients to participate?

- How do you interpret the results of patients’ PROMs and how would you discuss the results with your patients?

- Are you well informed on all aspects on how to use PROMs in practice? Any missing links?

3. Questions associated with the organization (centre, hospital, group/team; possibly also the broader work environment including colleagues elsewhere) (MIDI determinants 19, 23, 24, 26, 27, 28)

- What support is offered to you regarding the use of PROMs in your centre? By management, other professionals or whatever?

- How do you regard the time needed to use PROMs? Do you see any obstacles, apart from the normal lack of time?

- Which facilities do you have such as equipment, materials or IT support to implement PROMs? Is anything missing that can be seen as an obstacle in your daily practice?

- What other obstacles, in addition to the implementation of PROMs, do you experience in your work environment that affect the use of PROMs?

- How easy is it for you to find information in your work environment about using PROMs?

- What feedback is provided to you about the progress with PROM implementation? What do you expect from this feedback?

General question: What other comments do you have?
