# Supplementary material for: Facing the challenges of PROM implementation in Dutch dialysis care: Patients’ and professionals’ perspectives
Source: PLoS One. 2023 May 15;18(5):e0285822. doi: 10.1371/journal.pone.0285822 (PMC10184911; doi:10.1371/journal.pone.0285822)
Supplement: S3 File — (DOCX) [file pone.0285822.s003.docx]

**S3** **COREQ checklist**

**Facing the challenges of PROM implementation in Dutch dialysis care: patients’ and professionals’ perspectives**

**Consolidated criteria for reporting qualitative studies (COREQ): 32-item checklist**

Developed from:

Tong A, Sainsbury P, Craig J. Consolidated criteria for reporting qualitative research (COREQ): a 32-item checklist for interviews and focus groups. *International Journal for Quality in Health Care*. 2007. Volume 19, Number 6: pp. 349 – 357

| **No. Item** | **Guide questions/description** |  | **Section and page #** |
| --- | --- | --- | --- |
| **Domain 1: Research team and reﬂexivity** |  |  |  |
| *Personal Characteristics* |  |  |  |
| 1. Interviewer/facilitator | Which author/s conducted the interview or focus group? | WS | Methods (page 6) |
| 2. Credentials/team | What were the researcher’s credentials? E.g. PhD, MD | WS: MSc; KA: PhD/Ir. MdeJ: PhD/MD; YM: PhD; MH: PhD/MD. | Title page (page 1) |
| 3. Occupation/team | What was their occupation at the time of the study? | WS is currently a PhD candidate; KA is full professor health management research; MdeJ is nephrologist; YM is psychologist senior researcher; MH is full professor and nephrologist. MdeJ, YM en MH all work in different University Medical Centres. | Title page (page 1) |
| 4. Gender | Was the researcher male or female? | WS, KA, MH male; MdeJ, YM female | N/A |
| 5. Experience and training | What experience or training did the researcher have? | WS has three academic degrees and followed a two-year course on academic research including qualitative research prior to PhD candidacy; KA, MdeJ, YM and MH all have extended experience in conducting research projects employing qualitative methodologies. All have supervised PhD candidates. | N/A |
| *Relationship with participants* |  |  |  |
| 6. Relationship established | Was a relationship established prior to study commencement? | The interviewer had no prior relationship with any of the interviewees. MH selected professionals and knew most of the doctors who were interviewed professionally. | Methods (page 6) |
| 7. Participant knowledge of the interviewer | What did the participants know about the researcher? e.g. personal goals, reasons for doing the research | All participants were informed about the research prior to the interview and signed an informed consent form with a summary of the research goals. No personal goals were shared. | Methods (page 6) |
| 8. Interviewer characteristics | What characteristics were reported about the inter viewer/facilitator? e.g. Bias, assumptions, reasons and interests in the research topic | Interviewees were informed during the interview that the interviewer was a former dialysis patient. | Methods (page 7) |
| **Domain 2: study design** |  |  |  |
| *Theoretical framework* |  |  |  |
| 9. Methodological orientation and Theory | What methodological orientation was stated to underpin the study? e.g. grounded theory, discourse analysis, ethnography, phenomenology, content analysis | Qualitative inductive analysis. The Measurement of Determinants of Innovations (MIDI) was used in developing the interview protocol. | Methods (page 6) |
| *Participant selection* |  |  |  |
| 10. Sampling | How were participants selected? e.g. purposive, convenience, consecutive, snowball | For centres, recent early PROM implementation experience was required. Within the centres patients were selected on basis of convenience and willingness to share their thoughts. | Methods (page 6-7) |
| 11. Method of approach | How were participants approached? e.g. face-to-face, telephone, mail, email | Personal contact by email or phone. Centre staff served as intermediaries to contact patients. | Methods (page 6) |
| 12. Sample size | How many participants were in the study? | 13 professionals and 14 patients were interviewed. | Results (page 8) |
| 13. Non-participation | How many people refused to participate or dropped out? Reasons? | No one refused. | Results (page 8) |
| *Setting* |  |  |  |
| 14. Setting of data collection | Where was the data collected? e.g. home, clinic, workplace | All interviews with professionals were conducted in a hospital. Patients were interviewed at home, during dialysis in a centre, outside the hospital or by phone. | Results (page 8) |
| 15. Presence of non-participants | Was anyone else present besides the participants and researchers? | On three occasions, a family member was present. During dialysis, other patients and nurses were present. Nobody intervened during an interview. | Results (page 8) |
| 16. Description of sample | What are the important characteristics of the sample? e.g. demographic data, date | Professionals: 10 female, 3 male. 6 doctors, 6 nurses, 1 secretary. Patients: 3 female, 11 male. Patients’ ages ranged from 42 to 85. Time on dialysis varied from 6 months to 21 years. | Results (page 8) |
| *Data collection* |  |  |  |
| 17. Interview guide | Were questions, prompts, guides provided by the authors? Was it pilot tested? | Interview protocol was prepared and discussed among the authors. It was pilot tested with a doctor, a nurse and a patient. | Methods (page 6) |
| 18. Repeat interviews | Were repeat interviews carried out? If yes, how many? | No | N/A |
| 19. Audio/visual recording | Did the research use audio or visual recording to collect the data? | Interviews were audio recorded. | Methods (page 7)  Results (page 8) |
| 20. Field notes | Were ﬁeld notes made during and/or after the interview or focus group? | No | N/A |
| 21. Duration | What was the duration of the inter views or focus group? | Professionals: average 42 minutes (from 29 to 51 mins)’. Patients: average 29 minutes (from 13 to 51 mins). | Results (page 7) |
| 22. Data saturation | Was data saturation discussed? | Yes. | Methods (page 8) |
| 23. Transcripts returned | Were transcripts returned to participants for comment and/or correction? | No. For pilot interviews summaries were sent back. | Methods (page 6) |
| **Domain 3: analysis and ﬁndings** |  |  |  |
| *Data analysis* |  |  |  |
| 24. Number of data coders | How many data coders coded the data? | WS coded all interviews. Six interviews were also coded by KA and MdeJ. The developing themes were intensively discussed. | Methods (page 7) |
| 25. Description of the coding tree | Did authors provide a description of the coding tree? | Yes, both a coding tree and themes were generated. | Available on request. |
| 26. Derivation of themes | Were themes identiﬁed in advance or derived from the data? | Themes were derived from the data. | Methods (page 7) |
| 27. Software | What software, if applicable, was used to manage the data? | Atlas.ti 8.2 | Methods (page 8) |
| 28. Participant checking | Did participants provide feedback on the ﬁndings? | No | N/A |
| *Reporting* |  |  |  |
| 29. Quotations presented | Were participant quotations presented to illustrate the themes/ﬁndings? Was each quotation identiﬁed? e.g. participant number | Yes, quotations are presented to illustrate the themes/findings, and each quotation is identified with an anonymous participant identifier. | Results (page 8 – 25) |
| 30. Data and ﬁndings consistent | Was there consistency between the data presented and the ﬁndings? | Yes, there is consistency between the data presented and the findings. | Discussion (page 25-31) |
| 31. Clarity of major themes | Were major themes clearly presented in the ﬁndings? | Yes, major themes are clearly presented in the Results section using specific sections for each theme. | Results (page 8 -25) |
| 32. Clarity of minor themes | Is there a description of diverse cases or discussion of minor themes? | No | N/A |
