# Supplementary material for: Facing the challenges of PROM implementation in Dutch dialysis care: Patients’ and professionals’ perspectives
Source: PLoS One. 2023 May 15;18(5):e0285822. doi: 10.1371/journal.pone.0285822 (PMC10184911; doi:10.1371/journal.pone.0285822)
Supplement: S4 File — (DOCX) [file pone.0285822.s004.docx]

**S4 Illustrating quotes and corresponding MIDI-determinants**

| **Barriers** | **Subgroups** | **Illustrating quotes** | **Corresponding MIDI -determinants** [1] |
| --- | --- | --- | --- |
| Barrier 1: Patient indifference to PROMs | Lack of urgency | - I filled in the questionnaire because they asked me to, but we have the opportunity to speak with the doctor or head nurse every Monday. (p) | Personal benefits/drawbacks (8) |
|  | Questionnaire fatigue | - The problem we have is that our patients are overwhelmed by all these questionnaires. They suffer from questionnaire fatigue. (d) | Relevance for client (7) |
|  | Patient characteristic and trust | - For a few weeks now, I have a new doctor. My early experiences don’t encourage me to be more open. (p) | Patient cooperation (12) |
| Barrier 2: Scepticism on the benefits of aggregated PROM data | Doubts: are comparisons useful? | - Whether my patients are doing better or worse than those in a hospital 100 kms from here? I don’t think that is relevant. (d) - I’m not interested in PROMS as quality indicators. I have my own personal support here and I feel at home here. (p) | Relevance for client (7);  Personal benefits/drawbacks (8) |
|  | Doubts: are comparisons feasible with large case-mix differences? | - Our mortality rates are rather high. (d) - I believe that if you make nationwide comparisons between centres, you should declare the academic hospitals as a special group and, even within them, there are differences. (d) | Outcome expectations (9) |
|  | Doubts: do aggregated PROMs reveal differences in HRQOL? | - Suppose we find a higher quality of life in medium-sized municipality A as compared to patients in a densely populated urban city environment B. So what? (d) | Knowledge (17) |
|  | Doubts: the difficulties in following patients over time | - Our patients’ physical condition may deteriorate severely over time and still their quality of life score remains on the same high level. (n) | Observability (6) |
| Barrier 3: Limited treatment options open to doctors | Are dialysis doctors motivated? | - Not every doctor invests in the annual extended consultation with their patient. … To put it bluntly, some nephrologists see dialysis treatment as a tick-box exercise. (d) | Professional obligation (10) |
|  | Are doctors able to adequately intervene? | - I think PROMs can be a problem for some doctors who will find it difficult to discuss complaints that they cannot do anything about. … Even to me this is a bit frustrating. (d) | Self-efficacy (16) |
|  | Patients’ preferences and protocol conflict | - We are assessed on achieving good lab results, but maybe the patient only wants to undergo dialysis twice a week and this improves his quality of life. In terms of the visitation review, we are doing a bad job ­– but the patient is happier. (n) | Compatibility (5) |
| Barrier 4: Organizational and operational issues | Procedural growing pains | - We currently combine it with the annual consultation, and it cannot be right that, a second time, the PROM is not discussed with the patient. ... That still puzzles us. ... Maybe we just have to decide to keep it to once a year. (n) | Procedural clarity (1) |
|  | Not only the doctor, but the whole team is needed | - It doesn’t take much time from me, but the secretary staff and the nurses, yes it takes them extra time and, nowadays, their workload is already quite high. (d) - A big dialysis centre with more locations, well … then you would need a more structured approach and you have to train all those teams. (d) | Staff capacity (21) |
|  | Nursing staff: pain but no gain | - I have never noticed that doctors asked us as nurses how we see things. (n) | Social support (13) |
|  | Interference from external and internal turmoil | - Especially in these times with a lot of turmoil [a relocation of the department; WS] I believe that good projects can die because they are started at the wrong moment. (d) | Unsettled organisation (26) |
|  | Inevitable IT nonalignment | - At the moment it brings additional paperwork, if I want PROM results in the EHR I have to retype them so to speak. (d) | Complexity (4) |
| **Facilitators** |  |  |  |
| Facilitator 1: Professional involvement and patient support | Involving professionals as implementers | - It was decided [PROM implementation; WS]. We just had to implement it. Because I am studying to become a quality assurance nurse I thought it would be perfect to choose this as a topic for my thesis. (n) | Coordinator (25) |
|  | Best practices and lessons from previous experience | - Yes, that helps of course, because it already felt familiar. The procedure was already known, so in fact not much has changed. (n) | Procedural clarity (1) |
|  | Organizing support for patients with low health literacy skills | - In particular, the patient who finds this difficult is the patient that also has difficulties expressing himself to the doctor. … (d) - At home I feel more at ease. (..) I do know how to use a computer but I’m not a freak. Settings are always a bit different and I’m more comfortable when I use my own computer. (p) | Patient cooperation (12);  Material resources and facilities (24) |
| Facilitator 2: A growing understanding of the use of PROMs | Learning to assess patient responses | - I think that’s a matter of experience. An item score of 15 or 30 means nothing to me but, at some moment, when you use questionnaires more often, then you master it yourself. (d) | Self-efficacy ( 16)  Knowledge (17) |
|  | The power of using one standard PROM set in dialysis | - It would be nicer if we all used the same questionnaire in haemodialysis and other CKD treatments, used it in the same way and built experience in the same way. (d) | Subjective norm (15) |
|  | Openness to share PROM experiences | - I always say, I’m learning from it, but also for another. (p) - We have to share these experiences in national task groups and at congresses. (d) | Patient cooperation (12)  Professional obligation (10) |
| Facilitator 3: Quick gains from using PROMs | Easy-to-use product | - I think they are very complete. (..) And also I feel they are easy to fill in. (n) - I think the list was extensive, but it wasn’t difficult. (p) | Completeness (3) Complexity (4) |
|  | Receiving instant feedback | - I found it useful. I already knew I’m physically not in good shape. … I mean, you always talk about it, and now it’s crystal clear on paper. (p) - The beauty is, also for the patient, that they get their report straight away, all in colours, which is very convenient. (n) | Patient satisfaction (11) |
|  | PROM as a practical tool and time saver | - I can immediately focus on the complaints that patients have reported, which is a great opening for further discussion. It doesn’t cost any time and I can directly aim to talk about relevant complaints. (d) | Compatibility (5) |
|  | Easy handling and better consultations | - I just have to press ‘save as’ to save the PROM pdf file from Nefrovisie-Renine in Diamant. … A matter of only a few small steps. (s) | Complexity (4) |
| Facilitator 4: A clear ambition on patient care | A shared view on patient involvement | - It’s another way of how we work, another way of gathering information and getting different information. Not just medical, but also psychosocial. Yes, I believe this is very important. (n) - This is of course a topical issue nationwide, the whole issue that patients should have more say in their treatment. In many professional groups, you notice that PROMs are growing in importance. In my opinion this was not yet the case five years ago. (d) | Professional obligation (10) |
|  | Clear leadership | - There was no choice whether personnel would accept it or not. So, we are just going to do this. Some people see the benefits, others don’t and consider it to be nonsense. Well, come on. It just has to be done. (n) | Formal ratification by management (19)  Coordinator (25) |
|  | Management support | - We [dialysis centre; WS] are an island within the hospital. We do something because we think it is important.. (n) | Formal ratification by management (19) |

(p) = patient

(d) = doctor

(n) = nurse

(s) = secretary

1. Fleuren MAH, Paulussen TGWM, Dommelen P, Buuren S Van. Towards a measurement instrument for determinants of innovations. Int J Qual Heal Care. 2014;26(5):501–10.
