# Supplementary material for: Facing the challenges of PROM implementation in Dutch dialysis care: Patients’ and professionals’ perspectives
Source: PLoS One. 2023 May 15;18(5):e0285822. doi: 10.1371/journal.pone.0285822 (PMC10184911; doi:10.1371/journal.pone.0285822)
Supplement: Attachment — Submitted filename: Response to reviewers.docx [file pone.0285822.s005.docx]

Dear Academic Editor, Sabina De Rosis, PhD

Many thanks for your response to our submission ID PONE-D-21-17498, entitled "Facing the challenges of PROM implementation in Dutch dialysis care: patients’ and professionals’ perspectives".

We are very grateful to you and the reviewers for the insightful comments and we have addressed all the comments in our revised manuscript. We genuinely feel that the revised manuscript has improved. We have highlighted all changes in our revised manuscript by using track changes with a red coloured text.

We look forward to your response.

Yours faithfully,

also on behalf of my co-authors,

Wim Sipma MSc MBA

Erasmus University Rotterdam

The Netherlands

|  | **REVIEWER 1** (Please read below ‘LN’ as ‘line number’) |
| --- | --- |
| Comment | *Dear authors,*  *Thank you for the opportunity to review your manuscript on this relevant topic.*  *The topic of this study, identifying barriers and facilitators in the PROM implementation, is relevant as PROMs are currently widely implemented in clinical practice and the voltage drop from well-organized research setting to clinical practice described before, and we need knowledge exchange to learn about an optimal implementation.*  *However, I do have some major concerns that need to be addressed. The paper is very extensively written with a lot of details, so that a main message does not clearly emerge. Details about dialyses, the PROM set (as the development is described in another paper), or the interviews should be for example be limited. The paper can be improved by being more concise, omitting side paths and by clearly pointing out the results. On the other hand, the authors do not provide information about how the intervention (use of PROMs) is implemented in the different centers and when the PROMs are completed and discussed in their clinical pathway (e.g. with the physician during the yearly visit? Not with the nurses? And how this could influence the opinion of patients and physicians. Without this information the results are hard to interpret.*  *In addition, it is important to place this study more in light of the existing literature. Finally, I have some doubts about the inclusions of patients in this study. I think patients who never completed a PROM or patients that cannot remember completing PROMs should be excluded or should be analysed in separate analysis as they opinion is biased. I think the authors should reconsider to write the outcomes within the context of the determinant’s framework of Fleuren (as they used the MIDI questionnaire as basis for their interviews).* |
| Response | We thank the reviewer for this positive feedback. We understand the reviewer’s concerns. The reviewer expresses five major concerns. Below the outline of our response.   a. In order to make the manuscript more concise we deleted details about dialysis, the PROM set and the interviews, including data collection and data analysis. We agree that hereby we can gain more focus on our main message. b. We understand that the reviewer has questions on implementation in the different centres. We adjusted the manuscript explaining that the dialysis centres are independent on how, when and if they implement PROMs. This results in different approaches on when and with whom PROM results are discussed. We also notice that during the early implementation, dialysis centres are learning and reconsider the chosen process. c. We agree that some extra background on literature may be needed. Therefore we embedded our research more in the existing literature for which we gratefully used the reviewer’s suggested references (Gibbons, Hansen, Mejdahl, Trillingsgaard, Lapin). d. We understand the reviewer´s concerns about inclusion criteria. However, we strongly feel that patients that cannot remember the PROM questionnaire, but did fill in the PROM questionnaire, should be included. We argue that their answers, like ‘PROMs are just one of many questionnaires´, are relevant information for implementers. However, we excluded the two patients who did not have any experience with PROMs at all. We explain this choice in the manuscript. e. As to the reviewer’s suggestion to write the outcomes by following the MIDI determinants, we explain why we have chosen a different path. We did consider to follow the MIDI determinants when writing the manuscript, however we concluded that valuable information would be lost.  In the following we will go into the details why we made this choice.   We hope our responses are satisfactory and meet the reviewer’s expectations. |
|  |  |
| Abstract 1. | *PROMs are not necessarily discussed by the doctor, but this can also be done by a nurse, psychologist or other clinician.* |
| Response | We thank the reviewer for noticing this. We changed ´doctor´ in ´clinician´. The sentence now reads as follows:   - Patient Reported Outcome Measures (PROMs) are increasingly used in routine clinical practice to facilitate patients in sharing and discussing health-related topics with their clinician. (LN 26)   We also changed ‘doctor’ into ‘clinician’ in other sentences in the manuscript when appropriate. |
|  |  |
| Introduction 1. | *A clear structure is missing in the introduction. I would start the introduction with the importance of using PROMs – reasons why the implementation of PROMs is difficult – explanation PROM set Dutch dialysis centers + aim of the study.* |
| Response | We thank the reviewer for his comment. We agree that the structure could be more clear. To do so we followed his suggestion and we changed the introduction accordingly.  The first paragraph now reads as follows:  - Quality measures in healthcare have long been focused on care processes and clinical status such as objective outcomes (e.g. survival rates) and have historically been dominated by the perspectives of healthcare professionals. [1,2] However, these measurements only partially reflect the value of healthcare as perceived by patients. Patients are in particular interested in healthcare outcomes that matter to them personally such as functional status and quality of life. [1] To measure value of healthcare as perceived by patients, patient-reported outcome measures (PROMs) are increasingly used in routine clinical practice. (…) (LN 54)  The second paragraph starts at LN 68:  - However, these potential benefits are not easily achieved and are hindered by two issues. First, the implementation of PROMs in healthcare settings faces several barriers including patient and physician scepticism about practicability, time constraints, fear of added work, lack of training, administrative burden on patients and staff and a lack of organizational support [3,11–14] that result in a slow dissemination and use of PROMs.  The third paragraph describes end-stage kidney disease in general. Details on the Dutch dialysis set can be found in the Methods section, subsection ‘Setting’. The third paragraph starts with:  - Our study focuses on the introduction of PROMs in the patient group with end-stage kidney disease (ESKD). The worldwide population of patients that need renal replacement therapy is estimated at over two million [2,3], while dialysis treatment incurs high healthcare costs and places a large burden on the health-related quality of life (HRQOL) of patients. [24] (LN 81)  Starting at LN 102 the aim of the study is described:  - The aim of this study is to understand the barriers and facilitators that both patients and professionals, as primary users of the dialysis PROM set, encounter during the early phase of its implementation and the challenges they face in realizing the claimed benefits of PROMs. Our findings can contribute to a better understanding of the operationalization of PROMs in daily practice, which may increase the likelihood of their sustainable use. |
| Introduction 2. | *There are quite a few studies done on the experiences of patients with the use / implementation of PROMs. It is valuable to give a brief description of the results of these studies (what barriers and facilitators are found in these studies?)* |
| Response | We thank the reviewer for this comment. We agree that an overview of existing literature would give more background to our study. We have read the suggested references and found them very useful. Especially the papers by Hansen (2019) en Mejdahl (2016, 2018) interested us, because of their close interaction with patients.  The paragraph now reads as follows, starting at LN 68:  - First, the implementation of PROMs in healthcare settings faces several barriers including patient and physician scepticism about practicability, time constraints, fear of added work, lack of training, administrative burden on patients and staff and a lack of organizational support [3,11–14] that result in a slow dissemination and use of PROMs. Also, sometimes patients do not complete PROMs because of simply forgetting or loss of motivation. [15]  This paragraph continues at LN 74:  - (…) For instance, recent studies in a variety of chronic diseases found that patients experienced hardly any, or none at all, advantages of completing PROMs [19–21] In addition a Cochrane systematic review concluded that PROM completion makes no or little difference to patients regarding their perception of health and social functioning. [18] In contrast, positive results are also found, for instance in a quantitative study that reported a positive influence of PROMs on patients’ self-control. [22] In summary, there is ambiguous evidence that the use of PROMs helps to improve care processes from the patient´s perspective. [8,23] |
| Introduction 3. | *This study aims to describe the barriers and facilitators of PROM implementation, but this paper is only limited to the experience of patients and professionals. So, please narrow the aim. Or is it opinion, as patient not using PROMs are also included?* |
| Response | We thank the reviewer for noticing this. Our aim was to do research on barriers and facilitators of PROM implementation from the perspectives of key users in clinical practice. Therefore, we included healthcare professionals, especially physicians and nurses, and patients and we excluded other stakeholders as hospital management and health insurance companies. To accommodate the reviewer’s comments we explained this in the revised manuscript, thus narrowing the scope as we also described above, sub 1. We narrowed the aim:  - The aim of this study is to understand the barriers and facilitators that both patients and professionals, as primary users of the dialysis PROM set, encounter during the early phase of its implementation and the challenges they face in realizing the claimed benefits of PROMs. (LN 102) |
| Introduction 4. | *The newest systematic review (Cochrane) on the effect of PROMs is missing (Gibbons, 2021) in the literature.* |
| Response | We thank the reviewer for bringing this valuable review to our attention. The systematic review was not yet available at the time of our submission. We have added the review to the introduction as can be seen above, sub 2. |
|  |  |
| Methods 1. | *The section ‘Setting’ is very extensive and not all information is relevant for this paper, especially the first part about the treatment. Please shorten this section.* |
| Response | We thank the reviewer for this observation. We agree that the section ‘Setting’ is too extensive. We have shortened the section by omitting details and we now use a more compact style of writing. To accomplish this, we now focus on the setting and omitted details on dialysis treatment. The opening sentence is rephrased to:  - In October 2018, a dialysis PROM set was introduced in the Netherlands. Dialysis centres are encouraged to use the set, however every centre is independent on how, when and if they introduce PROMs in their centre.(LN 113) |
| Methods 2. | *Section ‘Setting’ - The authors mention that an earlier pilot study showed a highly differentiated pattern among centers, with patient response rates to the PROM questionnaire varying from 6 to 71%, with an average of 24% (line 125-127). What are the reasons for this huge differences in response rate?* |
| Response | We thank the reviewer for asking this question. The article of Van der Willik (2021) to which we refer in the manuscript, explains this by noticing that ‘response rates were highest in centres where professionals indicated that they had put a lot of effort into informing and inviting patients’. This was confirmed during our interviews with clinicians. We now supply information on this in the revised manuscript, starting at LN 123:  - The pilot study showed a highly differentiated pattern among centres, with patient response rates to the PROMs questionnaire varying from 6% to 71%, with an average of 24%. The highest response rates were found in centres with high engagement of professionals.. |
| Methods 3. | *Please explain how the PROMs are implemented. This is interesting in relation to the aim. Are there different aspects of the implementation? The frequency of administration of PROMs? Please provide information about how the feedback is organized?* |
| Response | We thank the reviewer for the opportunity to clarify this. We agree that these questions are relevant in relation to the aim. To accommodate the reviewer’s questions, we elaborated on this in the revised version of the manuscript. In the Methods section we explain that we selected dialysis centres with the criterion that they were in the initial phase of implementation. We also describe that dialysis centres are free to decide how and when they implement the PROM set, see our rephrased sentence above, sub 1. Our comments in the Methods section include:  - The introduction of the PROMs is supported on a national level by Nefrovisie and addresses many of the considerations as described in the User’s Guide to Implementing Patient-Reported Outcomes Assessment in Clinical Practice. [40,41] This central support offers dialysis centres with a variety of implementation strategies – like central IT support, newsletters, meetings, an informative film for patients, leaflets and factsheets on the website of Nefrovisie. A part of the site is dedicated to PROMs and how to start with PROM implementation in a dialysis center and a frequency of two PROM questionnaires a year is recommended. (LN 128)  - The registry system Renine provides the centres with feedback on how many patients responded to the PROM questionnaire and the average DSI and SF-12 scores on the dialysis centre level. (LN 145)  In the Results section we describe that most centres chose to discuss PROM results between doctors and patient during the yearly extended consult. We also describe that centres are open to adjust this and reconsider their choices. |
| Methods 4. | *Please describe the PROMs on the different know aspects of PROM implementation (maybe using the PROM guidelines) and elaborate if and how this influence the results.* |
| Response | We appreciate the reviewer’s suggestion on this. We agree that a short overview of known aspects on this topic is appropriate in this paper. Therefore, we have added a dedicated paragraph on this in the introduction section, LN 68-80. As for the PROM dialysis setting, our field of study, we now made it more clear on how the implementation is supported on a national level by Nefrovisie. The paragraph on this starts at LN 128 (see also the response above, sub 3):  - The introduction of the PROMs is supported on a national level by Nefrovisie and addresses many of the considerations as described in the User’s Guide to Implementing Patient-Reported Outcomes Assessment in Clinical Practice. [40,41] |
| Methods 5. | *The sentence ‘we did not ‘fire’ questions at the interviewees’ is not very scientific. Please change this sentence. And delete some details about this process.* |
| Response | We thank the reviewer for this comment. We agree that this sentence is too informal. We changed the sentence accordingly:  - We had an open conversation with patients, letting them set the pace and allowing them to raise topics that they saw as important regarding the potential use and expectations of PROMs. (LN 177)  We also deleted some details about this process. |
|  |  |
| Results 1. | *I would like some more information about the participants. Please provide a table with a general description of the participants.* |
| Response | We thank the reviewer for this suggestion. To accommodate his suggestion we inserted Table 1. (LN 215) with the main characteristics of the patient-participants. Regarding the professional participants we add no further information. Partly because we have no information on the age of professionals or their working experience and also to protect privacy. |
| Results 2. | *Please provide some more information about the multi-person interviews? How many professionals were interviewed together?* |
| Response | We thank the reviewer for asking this question. We now share this information in detail in the Result section, LN 194:  - Three interviews with professionals were multi-person, one couple of two nephrologists and one couple of a nephrologist with a nurse. We also interviewed one group of three participants, of which two nurses and one secretary. |
| Results 3. | *From which 9 Dutch dialysis centers across the Netherlands did the included professionals came from? And again, please provide a table about how the PROMs are implemented. A suggestion is to use the PROs in clinical guidelines (ISOQOL).* |
| Response | We thank the reviewer for this question. We understand the need to be more specific about which hospitals were involved in the study. However, we cannot share this information fully. We guaranteed anonymity to all participants, including clinicians. Sharing the names of the centres would compromise this promise. In the manuscript we pseudonymized data as city names that were mentioned during the interviews. We therefore keep the information on a more general level, like ‘university’ and ‘regional’ hospitals. We kindly ask for the reviewer’s understanding.  The reviewer requests a table about how the PROMs are implemented. We now describe in the manuscript how the national support on implementation is organized and which strategies are used (Methods: LN 128 – LN 134).  We found that the local implementation in each centre is more iterative and centres are trying to find out what works best for them on in their specific context. To illustrate this we refer to LN 415:  - We currently combine it with the annual consultation, and it cannot be right that, a second time, the PROM is not discussed with the patient. ... That still puzzles us. ... Maybe we just have to decide to keep it to once a year.” (nurse 2).  Instead of presenting a table we chose for a comprehensive description of the support strategies. We feel this is an adequate way to explain what is happening in reality.  We hope that we accommodate the reviewer’s suggestion with this answer. |
| Results 4. | *The authors included patients in this study that did not yet completed PROMs and patients who could not remember having completed a PROM. I think these patients should be excluded, as you ask them questions about their experience with the implementation of PROMs. These patients cannot answer these questions.* |
| Response | We thank the reviewer for the opportunity to explain why we included patients who cannot remember the PROMs. We understand the difficulty of asking patients on their experiences when they cannot remember them but did fill in the questionnaire. However, we feel that these patients should be included since the aim of our study is to explore challenges during the initial implementation. As is illustrated in Supporting File 1, questions and protocol for patients, we also ask patients about their expectations. For instance, “Could you describe the possible important goals for you as a patient? What do you expect from the PROM questionnaires?”; “In your opinion, how do you experience communication with your doctor? To what extent do you expect improvements through the use of a questionnaire like PROM? Can you explain?” and “Why would you cooperate (or not) when you are offered the possibility of filling out PROM questionnaires?”. The questions make it possible to include patients that are not yet familiar with PROMs or have no active memory. We argue that this makes the results even more interesting, it represents how patients reflect on PROM use in the early stage of implementation. These results may be interesting for implementers.  In order to accommodate the reviewer’s concerns, we explain this in the revised manuscript in the Methods section (data collection).  - An inclusion criterion was that they had at least once filled in the dialysis PROM questionnaire. (LN 168) We exclude two patients who did not complete the PROM questionnaire from the results in the revised manuscript.  - We found that two of the patients we interviewed did not complete the PROM questionnaire and they were excluded from the analysis. Table 1. describes the main characteristics of the included patients. (LN 212)  We sincerely hope that this explanation of in- and excluding these patients meets the reviewer’s concerns. |
| Results 5. | *Describe differences between views of patients and healthcare professionals? I would describe the results of both groups separately.* |
| Response | We thank the reviewer for asking this question and the suggestion for a different approach. We discussed the exact same approach the reviewer is suggesting in an earlier draft of the manuscript. However, we concluded that some factors are intertwined and that the manuscript would be more readable and our message would be clearer when the perspectives of both patients and caregivers were reported more closely together. We feel that interviewees expressed the complex interplay that is happening in a dialysis context between patients, doctors and nurses.  Let us give an example of this to illustrate our view (LN 302): - However, doctors are reluctant to accept the results of any comparisons, as a doctor explained “Whether my patients are doing better or worse than those in a hospital 100 kms from here? I don’t think that is relevant.” (doctor 2). One patient stated his feeling on this as follows: “I’m not interested in PROMS as quality indicators. I have my own personal support here and I feel at home here.” (patient 5).  By our focus on reporting the different perspectives around themes, instead of reporting perspectives of patients’ and professionals separately, we take more into account what the data tells us and the close interaction that exists between patients, nurses and doctors and what is really happening in everyday life in a dialysis centre. In our opinion this helps to make the storyline more clear to the reader.  We hope we made our reasoning clear in the above and that the reviewer can agree with our choice. |
| Results 6. | *After reading section ‘Lack of urgency’ I was wondering what is the main aim of implementing PROMs in this specific group?* |
| Response | We thank the reviewer for asking this very elementary question. PROMs for this specific group were introduced because of the need, as discussed within the professional group of physicians, to extend the focus of treatment on HRQOL for dialysis patients. It is known from various studies that ESKD affects QoL seriously for patients. It is also known that symptoms related to HRQOL are not always noticed by physicians. This combination of factors led to the decision to select and introduce a PROM set for this specific group, a decision that was broadly supported by physicians and by the Dutch kidney patients association (NVN). The aim was to further improve communication between patients and caregivers and improve the quality of care. The process is described by Van der Willik (2019). However, we found that the value of PROMs is not clear to everyone at the beginning of the implementation. This might endanger the embedding of PROMs in the long run. We elaborate on this dilemma in the discussion paragraph. The reasons on forehand to introduce PROMs in the consulting room is certainly open for debate as is also shown by other studies that we refer to in the discussion section. For this, we gratefully used the references the reviewer brought to our attention. We now describe the reason for the PROM development in the introduction section as follows (LN 86):  - In addition, symptoms and disease burden are not always recognized by clinicians, where nurses seem to be more accurate than nephrologists. [26,27] Because of the potential benefits of PROMs and indications that PROMs can be of added value to patients with chronic kidney disease (CKD) a standard PROM set was developed for dialysis patients in Dutch renal care. [28,29]  And in the discussion section we address these results stating:  - Recent studies that included patients who at least responded twice to digitally available PROM questionnaires in the field of hematologic care and renal care had similar results on patient motivation, where patients did not consider PROMs as ‘a tool for them’. [19,21] We argue that it may take some time for both patients and doctors to learn how to use PROMs in their communication and create additional value for patients. Or, as Staniszweska et al. state, using PROMs and creating patient benefits is a matter of evolution, not revolution. [51] (LN 683) |
| Results 7. | *I would strongly suggest to describe the barriers and facilitators in the light of the determinants model (or other implementation science framework) to make it more readable and better to understand the result.* |
| Response | We thank the reviewer for proposing this possibility. We agree that readability and understandability of the results are an important, if not crucial, element of the manuscript. When analysing our results we found that a lot of valuable information we got from the interviews would be lost if we reported using the MIDI determinants model of Fleuren, although we used it as a framework for our interview protocol. In order to explain this choice to the reader in the revised manuscript (section data analysis) we included the following sentence (LN 182):  - During the process of analysing the results, we decided to switch to an inductive way of understanding. We found that the data we had collected were rich, and the use of inductive reasoning led to useful insights on both facilitators and barriers. As such, we consider the overarching themes of the barriers and facilitators that emerged to be a more accurate reflection of reality and we present the results accordingly, following the inductive themes and not the predefined MIDI determinants.  On the other hand, we do understand the reviewer’s suggestion. It could be helpful to the readers, who are interested in implementing issues associated with PROMs, to also read the results of the study in connection with the MIDI determinants. Therefore we added supporting file 4 (S4) where we present a table, as suggested by reviewer #2, with all barriers and facilitators, illustrative quotes and, to accommodate reviewer’s #1 suggestion, the appropriate MIDI determinants. We refer to LN 227 in the manuscript where we present S4:  - An overview of barriers and facilitators, together with illustrating quotes is presented in supporting file 4 (S4). In S4 we relate the MIDI determinants to corresponding barriers and facilitators.  To make the manuscript more readable we also shortened some paragraphs and used a more concise writing style.  We sincerely hope that our explanation and the additions in the manuscript together with supporting file 4 meets the reviewer’s comment. |
| Results 8. | *Section ‘Questionnaire fatigue’ – what kind of questionnaire do this patients complete even more?* *A short overview of all PROMs could be added to the table with information on the implementation.* |
| Response | According to the Dutch dialysis quality protocol, patient satisfaction surveys and regular questionnaires are performed, based for example on Consumer Quality (CQ) scores, short-from Picker experience questionnaire and Edmonton Frailty Scale (EFS). In addition, researchers find their way to dialysis centres to set out surveys on specialized topics on for instance experiences with taxi transport, diets, sleeping issues, restless legs, relationships and so forth. To complement this short overview, patients are physically examined on muscle strength and concentration issues. However, these examples are not mentioned during the interviews. It is just mentioned by doctors, nurses and patients that they are often confronted with questionnaires and inquiries on a broad range of health related issues. For this reason, we decided to mention this in the discussion section. It helps the reader to understand why questionnaire fatigue is an issue when introducing a new additional questionnaire as PROMs. We introduced the next sentences (LN 694):  - We note that according to the Dutch dialysis quality protocol patient satisfaction surveys and regular intake questionnaires like the yearly anamnesis are also performed. In addition, Consumer Quality (CQ) scores, short-form Picker experience questionnaire and Edmonton Frailty Scale (EFS) exist for dialysis patients. Patient experience measures are encouraged by the Dutch Ministry of Health, Welfare and Sports. [52] All this can lead to questionnaire fatigue, or loss of interest and motivation. |
| Results 9. | *Section ‘patient characteristics and trust’ – I go there once a year and always tell more there than here. It’s a matter of trust… Over there I have already had the same doctor for eight years. Here, it changes every time’ (266-267) – I don’t think this says something about the implementation of PROMs, or relate this to the use. Who should discuss the PROMs. Could you add some implementation strategies too?* |
| Response | We thank the reviewer for the opportunity of explaining this.  a. With regard to the first comment above, our argument is that patients may not always be complete or honest when answering PROMs questions. We now explain this in a new sentence (LN 277):  - We found that the willingness to share personal information, which is a part of PROM questionnaires, is also linked to the trust relationship between caregiver and patient. Low trust might hamper open communication between doctor and patient, (…)  b. With regard to discussing PROMs, the results show that dialysis centres chose to discuss PROMs between doctors and patients. This was illustrated during the interviews by stressing that PROM discussions were planned around the yearly extended consult, between doctor and patient. During the interviews we found that nurses play an important role coordinating the distribution of PROMs, but did not systematically discuss results with patients.  c. Finally, with regard to implementation strategies, in the section Results of the manuscript we highlight used strategies that were mentioned during the interviews. For instance, we refer to the following sentences:  - During some of the interviews, it was described how the coordinator, a healthcare professional, managed all the preparatory operations such as the distribution of PROMs, staff training and motivation in cooperation with the centre’s management team. The planning and distribution of PROMs support was arranged by a central point in the various dialysis centres, for instance by a secretary or support staff such as a quality manager or nurse. (LN 482)  - Respondents described several preparatory activities that could achieve a higher response rate. These involved preparing to help and support patients in completing the questionnaires, linking the annual anamnesis assessment that has some of the same questions with PROMs, providing material support such as tablets, and giving patients the freedom of choice as to where and when they respond, either at home or during dialysis. (LN 521)  We point out that our study focuses on the challenges that professionals and patients face to successfully implement PROMs, our aim is not to describe successful strategies. We hope that our answer meets the reviewer’s expectations. |
| Results 10. | *Barrier 2: Scepticism on the benefits of aggregation. The term ‘aggregation’ is not very clear in this sentence. Maybe you could change it to aggregated PROM data?* |
| Response | We thank the reviewer for bringing this to our attention. We follow the suggestion and improved the sentence by changing the phrase to ‘aggregated PROM data’. The new sentence is:  Scepticism on the benefits of aggregated PROM data (LN 289). |
| Results 11. | *Three sections are about the doubts of aggregated PROM data, maybe you can cluster these sections together. Or please explain why the authors emphasis this aspect of PROM use.* |
| Response | We thank the reviewer for the opportunity to better explain this. PROMs have the potential to improve quality of care and for this aggregated PROM data can be used. However, there are some doubts. We prefer the use of the subsections and feel this is justified because of the different reported issues. To make this more clear to the reader, we explain this more in detail in the revised manuscript. We therefore rephrased the introduction of the paragraph (LN 291):  - We describe them in four subsections that each highlight a different aspect of potential benefits from a different perspective: the usefulness of comparing aggregated PROM data according to patients and clinicians, the feasibility of comparisons according to clinicians, the limited possibilities of aggregated PROMs to reveal differences in HRQOL as an indicator of quality of care and the difficulties with PROMs of following patients’ wellbeing over time.  We hope this explanation meets the reviewer’s criteria. |
| Results 12. | *In section ‘Doubts: the difficulties in following patients over time’ the authors describe that completing PROMs once a year may hinder the effective monitoring of patients over time. Can you support this statement with data?* |
| Response | We thank the reviewer for this comment. We added some additional information to support the statement. The paragraph now reads as:  - Here, although Nefrovisie recommends a distribution twice a year of dialysis PROMs, we found that in practice PROMs were only distributed among in-centre haemodialysis patients once a year prior to their scheduled annual extended consultation with a doctor. This was because professionals felt that the yearly consultation was the right moment to discuss the PROMs with their patients, “We want to distribute the PROM prior to the extended consultation, which is once a year.” (doctor 5). This low frequency may hinder the effective monitoring of patients over time. (LN 346) |
| Results 13. | *Section ‘Patients’ preferences and protocol conflict – please explain more in depth why and how this is a barrier to the implementation of PROMs.* |
| Response | We thank the reviewer for the question to clarify this. We agree that we have not made this quite clear. We adjusted the beginning and the end of this paragraph explaining why we think this result is a potential barrier for implementation.  - Professionals also reported a potential conflict with existing medical protocols. PROMs intend to give the patient a voice and more saying in the way they are treated. However, patients’ preferences may be in conflict with existing medical protocols. (LN 386)  - Here, it is important to note that compliance with protocols is safeguarded through external visitations. As a result, nursing staff who are in many cases responsible for distributing PROMs and motivate patients to respond, might be reluctant to do so if they feel that PROM results may not lead to changes in treatment and that patient preferences are disregarded due to protocol restrictions. (LN 396) |
| Results 14. | *Not all facilitators are formulated as a facilitator, for example ‘Using a single PROM set in the nephrology and dialysis field.’* |
| Response | We thank the reviewer for noticing. We adjusted the text accordingly and changed the names of the facilitators if appropriate.  - The power of using one standard PROM set in dialysis (LN 547)  - Openness to share PROM experiences (LN 556)  - PROMs as a practical tool and time saver (LN 592)  - Facilitator 4: A clear ambition on patient care (LN 620) |
|  |  |
| Discussion 1. | *In the results the barriers are described first. In the discussion section you first mention the facilitators and then the barriers in the section doctors. Please keep it consequent throughout the whole paper.* |
| Response | We thank the reviewer for noticing. We corrected this and now keep it consequent. In the section doctors now barriers are mentioned first (LN 720) and facilitators subsequently (LN 748). |
| Discussion 2. | *In the discussion section it is mentioned that doctors did not experience scepticism on the use of PROMs in the consultation room. However, lack of urgency was mentioned in the results section. Please explain.* |
| Response | We thank the reviewer raising this issue. We agree that this might seem in contradiction. However, lack of urgency was reported from the perspective of patients (LN 237).  Doctors’ scepticism is described in literature as a potential barrier (LN 752), an element that is not confirmed by our interviewees. To clarify this we added an additional explaining sentence in the paragraph. This reads as follows (LN 757):  - We found no scepticism by doctors on the use of PROMs in the consulting room. Instead, doctors were curious about the impact of PROMs during consultations and were hoping that discussion of PROMs would lead to a better understanding about patients´ health related concerns. |
| Discussion 3. | *As the study (as well as the PROM implementation) was conducted in time of the COVID pandemic. Please elaborate on how this socio-political context potentially influenced the implementation.* |
| Response | We thank the reviewer for this comment. The COVID pandemic had some impact on the study.  The location of the interviews with patients was sometimes adjusted because of COVID.  - Interviews were located either at the patient’s home (n=5), during dialysis in the hospital (n=5), in a private room in the hospital (n=2), outside but close to the hospital (n=1) and by phone (n=1). The last two due to COVID limitations. (LN 204)  We also contacted a few dialysis centres for interviews that had postponed the introduction of PROM implementation due to COVID. However, these centres did not participate in the study. To accommodate the reviewer’s comment on this and because it might be relevant information for the reader, we made an explicit remark about COVID and participating dialysis centres in the Strengths and Limitations section (LN 836):  - Some centres could not participate in this study because of COVID restrictions and had to postpone the implementation process. |

|  | **REVIEWER 2 (**Please read below ‘LN’ as ‘line number’.) |
| --- | --- |
| Comment | The work of Sipma and colleagues is an interesting paper on barriers and facilitators of PROMs collection in Dutch dialysis centers in . The work is based on structured interviews to patients and healthcare professionals that were developed using the Measurement Instrument for Determinants of Innovation (MIDI) framework. The authors identified four barriers: patient´s indifference to PROMs, scepticism on the benefits of aggregation, the limited treatment options open to doctors and organizational issues such as mergers, organizational problems and renovations. They also describe four facilitators professional involvement and patient support, a growing understanding of the use of PROMs during the implementation, quick gains from using PROMs such as receiving instant feedback and contextual elements such as a shared view on patient involvement and management support. The authors provide an adequate reporting of their results using the COREQ checklist and providing the questionnaires used in the study. They adequately put their results in the context of the literature on PROMs implementation. English seems adequate for publication. |
| Response | We thank the reviewer for this positive feedback. In the following we answer the questions and we gratefully use the suggestions and comments for improvement of the manuscript. |
|  |  |
| Comment 1. | *I think that general consideration on dialysis in the setting paragraph (line 93-110) should be put in the introduction paragraph.* |
| Response | We thank the reviewer for this suggestion. We agree that this would be an improvement and more logical to the reader. We therefore moved this part of the text to the introduction section. The new paragraph starts at LN 81:  - Our study focuses on the introduction of PROMs in the patient group with end-stage kidney disease (ESKD). The worldwide population of patients that need renal replacement therapy is estimated at over two million [2,3], while dialysis treatment incurs high healthcare costs and places a large burden on the health-related quality of life (HRQOL) of patients. [24] |
| Comment 2. | *The description of the implementation of dialysis PROMs in Netherlands should be described only in setting not in introduction.* |
| Response | We thank the reviewer for this comment and we have combined this with comment 1. As a result the Introduction now describes the general information on dialysis and the Setting section focuses on the implementation, together with the background of the dialysis PROM set development process. We also clarify how the set was introduced and accepted as a standard in the Netherlands because we felt this needed some extra explanation. The new paragraph starts at LN 113:  - In October 2018, a dialysis PROM set was introduced in the Netherlands. Dialysis centres are encouraged to use the set, however every centre is independent on how, when and if they introduce PROMs in their centre. The set was developed in close cooperation with Nefrovisie, the Dutch quality institute for nephrology care, and the Dutch Association of Kidney Patients (NVN) and has been accepted as the standard set by all 95 dialysis centres in the Netherlands that together take care of 6,300 dialysis patients. |
| Comment 3. | *It is not clear the relationship between Renine, Diamant, hospital EHR. The authors should provide a figure illustrating the information flow between the different systems and actors. Clarify whether PROMs information is provided to patient, doctors or nurse in aggregated form or at single person level.* |
| Response | We thank the reviewer for this suggestion. We gladly clarify the interdependencies between the different systems. We describe the details in a new paragraph, starting at LN 135:   - A digital approach was chosen to minimize administrative burden, a known barrier in the implementation of PROMs [31] (…)   We also provide in this paragraph information on single or aggregated level and on patients, doctors or nurses.  To summarise this, we now include ‘Fig 1 Involved IT systems’ in the manuscript. The figure can be found at LN 144. |
| Comment 4. | *Please explain whether and how COVID-19 pandemics had impacted on this study and if the theme emerged during interviews.* |
| Response | We thank the reviewer for raising this issue. We describe the impact in two places in the manuscript. First the impact on locations of the interviews with patients, and second in the section Strengths and Limitations the impact on availability of dialysis centres to do research.  - Interviews were located either at the patient’s home (n=5), during dialysis in the hospital (n=5), in a private room in the hospital (n=2), outside but close to the hospital (n=1) and by phone (n=1). The last two due to COVID limitations. (LN 206)  - Some centres could not participate in this study because of COVID restrictions and had to postpone the implementation process. (LN 836) |
| Comment 5. | *Please define “early implementation” and why you have preferred this phase for your project, instead of more mature centers. Discuss if results in early implementation differ from results collected before implementation if data are available.* |
| Response | We thank the reviewer for the opportunity to explain this.  At the time of our research there were no dialysis centres in the Netherlands that had reached a mature level of PROM implementation. Together with Nefrovisie we decided to study this early implementation phase and we aimed to understand the challenges of this early phase. We also felt this was a unique opportunity to study this early phase because most studies, according to our knowledge, focus on results of PROM implementation and the strategies used. We added a paragraph in the Introduction section to clarify why we focus on early implementation and what, in this study, is defined as ‘early implementation’. The paragraph starts at LN 90:  - We focus this study on the early implementation of this PROM set in Dutch dialysis centres, where early implementation is defined as the phase where ‘the decision to introduce the new dialysis PROM set in the dialysis centre has been made and professionals are actually working on implementation in the centre’.  At LN 94: - We focus on early implementation because most studies on PROM implementation were conducted before the implementation process started, in the context of a pilot study or after it was fully implemented. [17,20,21,30–35]  Unfortunately data on results after implementation are not available. However results of the pilot study of PROMs in Dutch dialysis care are published. See: Routinely measuring symptom burden and health-related quality of life in dialysis patients: first results from the Dutch registry of patient-reported outcome measures, Van der Willik et al., Clinical Kidney Journal, 2021, vol. 14, issue 6, June 2021, pages 1535 – 1544; DOI: 10.1093/ckj/sfz192.  We hope this explanation meets the reviewer’s criteria. |
| Comment 6. | *Is there significant difference in the intervention in the different centers? (EHR integration is always absent? Proms are provide everywhere only once a year? Nurses are equally involved in all centers? All patients see their results?)* |
| Response | We thank the reviewer for asking these questions. All centres are free to choose how, when and if they introduce the dialysis PROM set. All can determine their own pace and adjust the process to their possibilities in time, staff and priorities. The interviewed centres have in common that they are early adopters. We feel this makes our study interesting, because no centre is already in a state of maturity of using PROMs. The challenges and barriers are ‘fresh’, and divers. We feel this might add to the knowledge on barriers and facilitators, also because we have interviewed both patients and professionals. As a result, not everyone has the same working processes, or have the same ideas on involvement of nurses and feedback to patients. To explain this setting more in the manuscript we have added some extra information on this. Paragraphs addressing the reviewer’s questions are:  - In October 2018, a dialysis PROM set was introduced in the Netherlands. Dialysis centres are encouraged to use the set, however every centre is independent on how, when and if they introduce PROMs in their centre. (LN 113)  - The introduction of the PROMs is supported on a national level by Nefrovisie and addresses many of the considerations as described in the User’s Guide to Implementing Patient-Reported Outcomes Assessment in Clinical Practice. [43,44] (LN 128) |
| Comment 7. | *Provide a table summarizing barriers and facilitators you have found with their relative subthemes with synthetic definition and significant quotes from participants.* |
| Response | We thank the reviewer for this suggestion. We gladly provide a table with barriers, facilitators and relative subthemes together with significant quotes from participants. The table is added to the manuscript as supporting file 4 (S4). As a response to reviewer #1 we also added to this table the most appropriate MIDI determinants. We added a paragraph in the Results section that refers to S4 (LN 227):   - An overview of barriers and facilitators, together with illustrating quotes is presented in supporting file 4 (S4). In S4 we relate the MIDI determinants to corresponding barriers and facilitators.   We feel that the presented subthemes are an accurate synthetic definition of the barriers and facilitators. In our opinion adding a supplementary definition might distract the reader from our storyline. Therefore we do not prefer to add an extra definition of barriers and facilitators.  We hope that the reviewer can agree with our argument. |
| Comment 8. | *In results you state the problem of EHR Integration but it is not commented further in results, please consider these references to further comment on this topic: (1.) Reported Outcome Measures to the Electronic Health Record for Comparative Effectiveness Research. J Clin Epidemiol. 2013 Aug;66(8 0):S12–20. (2.) Snyder C, and Wu, A.W. Users’ Guide to Integrating Patient-Reported Outcomes in Electronic Health Records [Internet]. Johns Hopkins University. 2017. Funded by Patient-Centered Outcomes Research Institute (PCORI); Available from: : http://www.pcori.org/document/users-guide-integrating-patient-reported-outcomeselectronic-health-records* |
| Response | We thank the reviewer for the interest in the EHR Integration issues. We gladly discuss this further in the manuscript and we have chosen to elaborate on this in the Discussion section. The dedicated paragraph starts at LN 779:  - Regarding integration of patient data, literature emphasizes the importance to integrate PROM data with the patient’s electronic health record to overcome practical barriers and to enable comparative effectiveness. [66] In our study we found that a hybrid model was chosen, with an external platform for data collection. [67] |
| Comment 9. | *In discussion please state if and how these results will be used for further development of PROMs in participating dialysis centers.* |
| Response | We thank the reviewer for this suggestion. We have added a new paragraph to the manuscript, named Lessons for practice. It starts at LN 820:  - To improve care from a patient´s perspective, long-term collecting of patient outcomes is necessary. We argue that our study shows that significant progress has been made to overcome practical barriers of PROMs, although some of them are still present. This has resulted in an easy-to-use PROM questionnaire for both patients and clinicians, a well-organised digital approach of collecting and distributing PROMs and a strong motivation of clinicians involved in the implementation. However, this not enough to get acknowledgement of patients. Today, patients do not recognize direct benefits of PROMs and to fill in the questionnaire they are mainly motivated by the argument that they are helping their doctor. In our opinion, the most crucial step forward now is to improve support interventions based on PROM results that patients report. When PROMs are used to improve patients’ lives, and this is clear to patients, then we feel confident that patient’s acknowledgement for the use of PROMs will follow. In summary, we suggest the next step is to close the gap between symptom awareness and symptom management.  In addition, we can announce that one of the authors is currently working on a program to develop interventions based on the use of PROM results in dialysis care for the benefit of patients. The project started in April 2022. (<https://nierstichting.nl/nieuws/2022/04/effectieve-behandelingen-voor-een-hogere-kwaliteit-van-leven/>) |
| Comment 10. | *Reference are not always consistent with PLOS standard (see https://journals.plos.org/plosone/s/submission-guidelines#loc-references). In the first reference the journal (BMC Nephrology) is missing and often published articles are referred as online articles.* |
| Response | We thank the reviewer for bringing this to our attention. We checked all the references and made the necessary adjustments. |
