# Supplementary material for: Facing the challenges of PROM implementation in Dutch dialysis care: Patients’ and professionals’ perspectives
Source: PLoS One. 2023 May 15;18(5):e0285822. doi: 10.1371/journal.pone.0285822 (PMC10184911; doi:10.1371/journal.pone.0285822)
Supplement: Attachment — Submitted filename: Response to reviewers revised manuscript 2023.docx [file pone.0285822.s006.docx]

Dear Dr. Sabina De Rosis,

Many thanks for your response to our submission ID PONE-D-21-17498, entitled "Facing the challenges of PROM implementation in Dutch dialysis care: patients’ and professionals’ perspectives".

We are very grateful to you and the reviewers for the comments on our revised manuscript. We have addressed the suggestions and remaining questions in our newly revised manuscript. We have highlighted the changes by using track changes with a red coloured text.

In addition, we would like to express our gratitude towards you for your commitment to monitor the review process.

We look forward to your response.

Yours faithfully,

also on behalf of my co-authors,

Wim Sipma MSc MBA

Erasmus University Rotterdam

The Netherlands

|  | **REVIEWER 2** |
| --- | --- |
| Comment | *The manuscript has improved since the previous version and the authors have answered all my questions.* |
| Response | We thank the reviewer for this positive feedback. We also thank the reviewer for his earlier comments that allowed us to improve the manuscript. |

|  | **REVIEWER 3** |
| --- | --- |
| Comment | *The data analysis lacks a section about the different aspects of trustworthiness of the study. In the discussion, the internal validity is addressed to some extent, but the manuscript lacks aspects of credibility, transferability, dependability, and confirmability.*  *- Related to trustworthiness of the study: how were the authors IM and MH (in addition to the main researchers WS, KA and MdJ) involved in conforming the themes identified in the study?* |
| Response | We thank the reviewer for this observation and we gladly clarify the trustworthiness of the study. All authors were fully involved during this study, including the study design and discussing the results and themes. The academic background and expertise of the authors is stated in the accompanying COREQ checklist, included in Supporting Information File 3.  To clarify the trustworthiness of the study in the manuscript we added the following paragraph in the section Data analysis section, line number 192.   - “To consider the trustworthiness of this study we refer to the elements credibility, dependability, transferability and confirmability. (48) To enhance the credibility of the data all authors were involved during the research from the study design to discussing the themes and interpret the results. To ensure dependability we described how we collected the data and how we analysed the transcriptions deploying an iterative process supported by coding software. With regard to transferability, although this study is focused on PROMs implementation in dialysis centres, the findings could well be used in other healthcare settings especially in chronic care management. Confirmability was assured by having critical discussions within the research team, continuous checking of concepts and exchanging information.”   Reference 48: Amir N, McCarthy HJ, Tong A. Qualitative Research in Nephrology: An Introduction to Methods and Critical Appraisal. Kidney360. 2021 Feb 22;2(4):737-741  We hope our response is satisfactory and meets the reviewer’s expectations. |
|  |  |
| Comment | *I could not find a statement about availability of data.* |
| Response | We thank the reviewer for this comment. We submitted the following data availability statement to PLOS ONE that will be published in the editors’ information that accompanies the publication of the study.   - “Data cannot be shared publicly because of confidentiality. For further information please contact the office of the Medical Ethics Review Board (METc) of the University Medical Center Groningen (UMCG), email metc@umcg.nl. Data are available upon request for researchers who meet the criteria for access to confidential data.“   We trust this is sufficient to inform the reviewer on data availability. |
