# Supplementary material for: Facing the challenges of PROM implementation in Dutch dialysis care: Patients’ and professionals’ perspectives
Source: PLoS One. 2023 May 15;18(5):e0285822. doi: 10.1371/journal.pone.0285822 (PMC10184911; doi:10.1371/journal.pone.0285822)
Supplement: Peer review history

# Peer review: Decision Letter 0

**DOI:** 10.1371/journal.pone.0285822.r001

**Authors:** Sabina De Rosis

4 May 2022

PONE-D-21-17498Facing the challenges of PROM implementation in Dutch dialysis care: patients’ and professionals’ perspectivesPLOS ONE

Dear Dr. Sipma,

Thank you for submitting your manuscript to PLOS ONE. After careful consideration, we feel that it has merit but does not fully meet PLOS ONE’s publication criteria as it currently stands. Therefore, we invite you to submit a revised version of the manuscript that addresses the points raised during the review process. Particularly, one of the reviewers has several major concerns that need to be addressed.The PLOS ONE criteria for publication includes the narrative of the study, its presentation in an appropriate and intelligible fashion. Please, revise your paper by selecting and limiting the details, assuring that the key messages arrive to the readers. Making the paper more concise on not necessary details can help you in deepening essential aspect of your research, such as how the intervention was implemented in the different centers and when the PROMs are completed and discussed, how this could influence the opinion of patients and physicians, ad so on. Both reviewers underlined that the study should be better framed into the existing literature. Finally, one reviewer has serious concerns about your criteria of patients' inclusion into the study (patients who never completed a PROM or patients that cannot remember completing PROMs): please revise reconsider to write the outcomes or better explain your decisions on the bases of the available evidence. I recommend to follow the indications of the two reviewers. In addition, you can find here the PLOS ONE’s publication criteria. Please submit your revised manuscript by Jun 18 2022 11:59PM. If you will need more time than this to complete your revisions, please reply to this message or contact the journal office at plosone@plos.org. When you're ready to submit your revision, log on to https://www.editorialmanager.com/pone/ and select the 'Submissions Needing Revision' folder to locate your manuscript file.

Please include the following items when submitting your revised manuscript:

- A rebuttal letter that responds to each point raised by the academic editor and reviewer(s). You should upload this letter as a separate file labeled 'Response to Reviewers'.
- A marked-up copy of your manuscript that highlights changes made to the original version. You should upload this as a separate file labeled 'Revised Manuscript with Track Changes'.
- An unmarked version of your revised paper without tracked changes. You should upload this as a separate file labeled 'Manuscript'.

If you would like to make changes to your financial disclosure, please include your updated statement in your cover letter. Guidelines for resubmitting your figure files are available below the reviewer comments at the end of this letter.

If applicable, we recommend that you deposit your laboratory protocols in protocols.io to enhance the reproducibility of your results. Protocols.io assigns your protocol its own identifier (DOI) so that it can be cited independently in the future. For instructions see: https://journals.plos.org/plosone/s/submission-guidelines#loc-laboratory-protocols. Additionally, PLOS ONE offers an option for publishing peer-reviewed Lab Protocol articles, which describe protocols hosted on protocols.io. Read more information on sharing protocols at https://plos.org/protocols?utm_medium=editorial-email&utm_source=authorletters&utm_campaign=protocols.

We look forward to receiving your revised manuscript.

Kind regards,

Sabina De Rosis, PhD

Academic Editor

PLOS ONE

Journal Requirements:

When submitting your revision, we need you to address these additional requirements.

1. Please ensure that your manuscript meets PLOS ONE's style requirements, including those for file naming. The PLOS ONE style templates can be found at 

https://journals.plos.org/plosone/s/file?id=wjVg/PLOSOne_formatting_sample_main_body.pdf and 

https://journals.plos.org/plosone/s/file?id=ba62/PLOSOne_formatting_sample_title_authors_affiliations.pdf

2. In your Data Availability statement, you have not specified where the minimal data set underlying the results described in your manuscript can be found. PLOS defines a study's minimal data set as the underlying data used to reach the conclusions drawn in the manuscript and any additional data required to replicate the reported study findings in their entirety. All PLOS journals require that the minimal data set be made fully available. For more information about our data policy, please see http://journals.plos.org/plosone/s/data-availability.

Upon re-submitting your revised manuscript, please upload your study’s minimal underlying data set as either Supporting Information files or to a stable, public repository and include the relevant URLs, DOIs, or accession numbers within your revised cover letter. For a list of acceptable repositories, please see http://journals.plos.org/plosone/s/data-availability#loc-recommended-repositories. Any potentially identifying patient information must be fully anonymized.

Important: If there are ethical or legal restrictions to sharing your data publicly, please explain these restrictions in detail. Please see our guidelines for more information on what we consider unacceptable restrictions to publicly sharing data: http://journals.plos.org/plosone/s/data-availability#loc-unacceptable-data-access-restrictions. Note that it is not acceptable for the authors to be the sole named individuals responsible for ensuring data access.

We will update your Data Availability statement to reflect the information you provide in your cover letter.

3. Please include captions for your Supporting Information files at the end of your manuscript, and update any in-text citations to match accordingly. Please see our Supporting Information guidelines for more information: http://journals.plos.org/plosone/s/supporting-information. 

[Note: HTML markup is below. Please do not edit.]

Reviewers' comments:

Reviewer's Responses to Questions

**Comments to the Author**

1. Is the manuscript technically sound, and do the data support the conclusions?

The manuscript must describe a technically sound piece of scientific research with data that supports the conclusions. Experiments must have been conducted rigorously, with appropriate controls, replication, and sample sizes. The conclusions must be drawn appropriately based on the data presented. 

Reviewer #1: Partly

Reviewer #2: Yes

**********

2. Has the statistical analysis been performed appropriately and rigorously? 

Reviewer #1: N/A

Reviewer #2: N/A

**********

3. Have the authors made all data underlying the findings in their manuscript fully available?

The PLOS Data policy requires authors to make all data underlying the findings described in their manuscript fully available without restriction, with rare exception (please refer to the Data Availability Statement in the manuscript PDF file). The data should be provided as part of the manuscript or its supporting information, or deposited to a public repository. For example, in addition to summary statistics, the data points behind means, medians and variance measures should be available. If there are restrictions on publicly sharing data—e.g. participant privacy or use of data from a third party—those must be specified.

Reviewer #1: Yes

Reviewer #2: Yes

**********

4. Is the manuscript presented in an intelligible fashion and written in standard English?

PLOS ONE does not copyedit accepted manuscripts, so the language in submitted articles must be clear, correct, and unambiguous. Any typographical or grammatical errors should be corrected at revision, so please note any specific errors here.

Reviewer #1: Yes

Reviewer #2: Yes

**********

5. Review Comments to the Author

Please use the space provided to explain your answers to the questions above. You may also include additional comments for the author, including concerns about dual publication, research ethics, or publication ethics. (Please upload your review as an attachment if it exceeds 20,000 characters)

Reviewer #1: Dear authors,

Thank you for the opportunity to review your manuscript on this relevant topic.

The topic of this study, identifying barriers and facilitators in the PROM implementation, is relevant as PROMs are currently widely implemented in clinical practice and the voltage drop from well organized research setting to clinical practice described before, and we need knowledge exchange to learn about an optimal implementation.

However, I do have some major concerns that need to be addressed. The paper is very extensively written with a lot of details, so that a main message does not clearly emerge. Details about dialyses, the PROM set (as the development is described in another paper), or the interviews should be for example be limited. The paper can be improved by being more concise, omitting side paths and by clearly pointing out the results. On the other hand, the authors do not provide information about how the intervention (use of PROMs) is implemented in the different centers and when the PROMs are completed and discussed in their clinical pathway (e.g. with the physician during the yearly visit? Not with the nurses? And how this could influence the opinion of patients and physicians. Without this information the results are hard to interpret.

In addition, it is important to place this study more in light of the existing literature. Finally, I have some doubts about the inclusions of patients in this study. I think patients who never completed a PROM or patients that cannot remember completing PROMs should be excluded or should be analyzed in separate analysis as they opinion is biased. I think the authors should reconsider to write the outcomes within the context of the determinant’s framework of Fleuren (as they used the MIDI questionnaire as basis for their interviews).

Abstract

1. The introduction of the abstract describes that PROMs are increasingly used in routine clinical practice to facilitate patients in sharing and discussing health-related topics with their doctor. I recommend broadening this term to clinicians or health care professionals, as PROMs are not necessarily discussed by the doctor, but this can also be done by a nurse, psychologist or other clinician.

Introduction

1. A clear structure is missing in the introduction. I would start the introduction with the importance of using PROMs – reasons why the implementation of PROMs is difficult – explanation PROM set Dutch dialysis centers + aim of the study.

2. There are quite a few studies done on the experiences of patients with the use / implementation of PROMs. It is valuable to give a brief description of the results of these studies (what barriers and facilitators are found in these studies?)

a. Staniszewska et al., The patient-Centered Outcome Research (2012)

b. Mejdahl et al., European Journal for Person Centered Healthcare (2016)

c. Mejdahl et al., Journal of Patient-Reported outcomes (2018)

d. Thestrup Hansen et al., Cander Nursing (2020)

e. Lapin et al., Quality of Life Research (2021)

f. Muilekom & Teela et al., Quality of Life Research (2021)

3. This study aims to describe the barriers and facilitators of PROM implementation, but this paper is only limited to the experience of patients and professionals. So, please narrow the aim. Or is it opinion, as patient not using PROMs are also included?

4. The newest systematic review (Cochrane) on the effect of PROMs is missing (Gibbons, 2021) in the literature.

Methods

1. The section ‘Setting’ is very extensive and not all information is relevant for this paper, especially the first part about the treatment. Please shorten this section.

2. Section ‘Setting’ - The authors mention that an earlier pilot study showed a highly differentiated pattern among centers, with patient response rates to the PROM questionnaire varying from 6 to 71%, with an average of 24% (line 125-127). What are the reasons for this huge differences in response rate?

3. Please explain how the PROMs are implemented. This is interesting in relation to the aim. Are there different aspects of the implementation? The frequency of administration of PROMs? Please provide information about how the feedback is organized?

4. Please describe the PROMs on the different know aspects of PROM implementation (maybe using the PROM guidelines) and elaborate if and how this influence the results.

5. The sentence ‘we dit not ‘fire’ questions at the interviewees’ is not very scientific. Please change this sentence. And delete some details about this process.

Results

1. I would like some more information about the participants. Please provide a table with a general description of the participants.

2. Please provide some more information about the multi-person interviews? How many professionals were interviewed together?

3. From which 9 Dutch dialysis centers across the Netherlands did the included professionals came from? And again, please provide a table about how the PROMs are implemented. A suggestion is to use the PROs in clinical guidelines (ISOQOL).

4. The authors included patients in this study that did not yet completed PROMs and patients who could not remember having completed a PROM. I think these patients should be excluded, as you ask them questions about their experience with the implementation of PROMs. These patients cannot answer these questions.

5. Describe differences between views of patients and healthcare professionals? I would describe the results of both groups separately.

6. After reading section ‘Lack of urgency’ I was wondering what is the main aim of implementing PROMs in this specific group?

7. I would strongly suggest to describe the barriers and faciliators in the light of the determinants model (or other implementation science framework) to make it more readible and better to understand the result.

8. Section ‘Questionnaire fatigue’ – what kind of questionnaire do this patients complete even more? A short overview of all PROMs could be added to the table with information on the implementation.

9. Section ‘patient characteristics and trust’ – I go there once a year and always tell more there than here. It’s a matter of trust… Over there I have already had the same doctor for eight years. Here, it changes every time’ (266-267) – I don’t think this says something about the implementation of PROMs, or relate this to the use. Who should discuss the PROMs. Could you add some implementation strategies too?

10. Barrier 2: Sceptism on the benefits of aggregation. The term ‘aggregation’ is not very clear in this sentence. Maybe you could change it to aggregated PROM data?

11. Three sections are about the doubts of aggregated PROM data, maybe you can cluster these sections together. Or please explain why the authors emphasis this aspect of PROM use.

12. In section ‘Doubts: the difficulties in following patients over time’ the authors describe that completing PROMs once a year may hinder the effective monitoring of patients over time. Can you support this statement with data?

13. Section ‘Patients’ preferences and protocol conflict – please explain more in depth why and how this is a barrier to the implementation of PROMs.

14. Not all facilitators are formulated as a facilitator, for example ‘Using a single PROM set in the nephrology and dialysis field.’

Discussion

1. In the results the barriers are described first. In the discussion section you first mention the facilitators and then the barriers in the section doctors. Please keep it consequent throughout the whole paper.

2. In the discussion section it is mentioned that doctors did not experience skepticism on the use of PROMs in the consultation room. However, lack of urgency was mentioned in the results section. Please explain.

3. As the study (as well as the PROM implementation) was conducted in time of the COVID pandemic. Please elaborate on how this socio-political context potentially influenced the implementation.

Reviewer #2: The work of Sipma and colleagues is an interesting paper on barriers and facilitators of PROMs collection in Dutch dialysis centers in . The work is based on structured interviews to patients and healthcare professionals that were developed using the Measurement Instrument for Determinants of Innovation (MIDI) framework. The authors identified four barriers: patient´s indifference to PROMs, skepticism on the benefits of aggregation, the limited treatment options open to doctors and organizational issues such as mergers, organizational problems and renovations. They also describe four facilitators professional involvement and patient support, a growing understanding of the use of PROMs during the implementation, quick gains from using PROMs such as receiving instant feedback and contextual elements such as a shared view on patient involvement and management support. The authors provide an adequate reporting of their results using the COREQ checklist and providing the questionnaires used in the study. They adequately put their results in the context of the literature on PROMs implementation. English seems adequate for publication.

Minor revision

1) I think that general consideration on dialysis in the setting paragraph (line 93-110) should be put in the introduction paragraph.

2) The description of the implementation of dialysis PROMs in Netherlands should be described only in setting not in introduction

3) It is not clear the relationship between Renine, Diamant, hospital EHR. The authors should provide a figure illustrating the information flow between the different systems and actors. Clarify whether PROMs information is provided to patient, doctors or nurse in aggregated form or at single person level.

4) Please explain whether and how COVID-19 pandemics had impacted on this study and if the theme emerged during interviews.

5) Please define “early implementation” and why you have preferred this phase for your project, instead of more mature centers. Discuss if results in early implementation differ from results collected before implementation if data are available.

6) Is there significant difference in the intervention in the different centers? (EHR integration is always absent? Proms are provide everywhere only once a year? Nurses are equally involved in all centers? All patients see their results?)

7) Provide a table summarizing barriers and facilitators you have found with their relative subthemes with synthetic definition and significant quotes from participants.

8) in results you state the problem of EHR Integration but it is not commented further in results, please consider these references to further comment on this topic:

1. Reported Outcome Measures to the Electronic Health Record for Comparative Effectiveness Research. J Clin Epidemiol. 2013 Aug;66(8 0):S12–20.

2. Snyder C, and Wu, A.W. Users’ Guide to Integrating Patient-Reported Outcomes in Electronic Health Records [Internet]. Johns Hopkins University. 2017. Funded by Patient-Centered Outcomes Research Institute (PCORI); Available from: : http://www.pcori.org/document/users-guide-integrating-patient-reported-outcomeselectronic-health-records

9) In discussion please state if and how theses results will be used for further development of PROMs in participating dialysis centers.

10) Reference are not always consistent with PLOS standard (see https://journals.plos.org/plosone/s/submission-guidelines#loc-references). In the first reference the journal (BMC Neprology) is missing and often published articles are referred as online articles.

**********

6. PLOS authors have the option to publish the peer review history of their article (what does this mean?). If published, this will include your full peer review and any attached files.

If you choose “no”, your identity will remain anonymous but your review may still be made public.

**Do you want your identity to be public for this peer review?** For information about this choice, including consent withdrawal, please see our Privacy Policy.

Reviewer #1: No

Reviewer #2: No

[NOTE: If reviewer comments were submitted as an attachment file, they will be attached to this email and accessible via the submission site. Please log into your account, locate the manuscript record, and check for the action link "View Attachments". If this link does not appear, there are no attachment files.]

While revising your submission, please upload your figure files to the Preflight Analysis and Conversion Engine (PACE) digital diagnostic tool, https://pacev2.apexcovantage.com/. PACE helps ensure that figures meet PLOS requirements. To use PACE, you must first register as a user. Registration is free. Then, login and navigate to the UPLOAD tab, where you will find detailed instructions on how to use the tool. If you encounter any issues or have any questions when using PACE, please email PLOS at figures@plos.org. Please note that Supporting Information files do not need this step.

# Author comment: Author response to Decision Letter 0

**DOI:** 10.1371/journal.pone.0285822.r002

1 Aug 2022

We are very grateful to the reviewers for the insightful comments and we have addressed all the comments in our revised manuscript. We genuinely feel that the revised manuscript has improved. We have highlighted all changes in our revised manuscript by using track changes with a red coloured text.

Attachment Submitted filename: Response to reviewers.docx Click here for additional data file.

# Peer review: Decision Letter 1

**DOI:** 10.1371/journal.pone.0285822.r003

**Authors:** Sabina De Rosis

2 Jan 2023

PONE-D-21-17498R1Facing the challenges of PROM implementation in Dutch dialysis care: patients’ and professionals’ perspectivesPLOS ONE

Dear Dr. Sipma,

Thank you for submitting your manuscript to PLOS ONE. After careful consideration, we feel that it has merit but does not fully meet PLOS ONE’s publication criteria as it currently stands. Therefore, we invite you to submit a revised version of the manuscript that addresses the points raised during the review process.

In particular, one reviewer asked for minor revisions, suggesting a more detailed description of the trustworthiness of your methodology and data.

Please submit your revised manuscript by Feb 16 2023 11:59PM. If you will need more time than this to complete your revisions, please reply to this message or contact the journal office at plosone@plos.org. When you're ready to submit your revision, log on to https://www.editorialmanager.com/pone/ and select the 'Submissions Needing Revision' folder to locate your manuscript file.

Please include the following items when submitting your revised manuscript:

- A rebuttal letter that responds to each point raised by the academic editor and reviewer(s). You should upload this letter as a separate file labeled 'Response to Reviewers'.
- A marked-up copy of your manuscript that highlights changes made to the original version. You should upload this as a separate file labeled 'Revised Manuscript with Track Changes'.
- An unmarked version of your revised paper without tracked changes. You should upload this as a separate file labeled 'Manuscript'.

If you would like to make changes to your financial disclosure, please include your updated statement in your cover letter. Guidelines for resubmitting your figure files are available below the reviewer comments at the end of this letter.

If applicable, we recommend that you deposit your laboratory protocols in protocols.io to enhance the reproducibility of your results. Protocols.io assigns your protocol its own identifier (DOI) so that it can be cited independently in the future. For instructions see: https://journals.plos.org/plosone/s/submission-guidelines#loc-laboratory-protocols. Additionally, PLOS ONE offers an option for publishing peer-reviewed Lab Protocol articles, which describe protocols hosted on protocols.io. Read more information on sharing protocols at https://plos.org/protocols?utm_medium=editorial-email&utm_source=authorletters&utm_campaign=protocols.

We look forward to receiving your revised manuscript.

Kind regards,

Sabina De Rosis, PhD

Academic Editor

PLOS ONE

Journal Requirements:

Please review your reference list to ensure that it is complete and correct. If you have cited papers that have been retracted, please include the rationale for doing so in the manuscript text, or remove these references and replace them with relevant current references. Any changes to the reference list should be mentioned in the rebuttal letter that accompanies your revised manuscript. If you need to cite a retracted article, indicate the article’s retracted status in the References list and also include a citation and full reference for the retraction notice.

[Note: HTML markup is below. Please do not edit.]

Reviewers' comments:

Reviewer's Responses to Questions

**Comments to the Author**

1. If the authors have adequately addressed your comments raised in a previous round of review and you feel that this manuscript is now acceptable for publication, you may indicate that here to bypass the “Comments to the Author” section, enter your conflict of interest statement in the “Confidential to Editor” section, and submit your "Accept" recommendation.

Reviewer #2: All comments have been addressed

Reviewer #3: (No Response)

**********

2. Is the manuscript technically sound, and do the data support the conclusions?

The manuscript must describe a technically sound piece of scientific research with data that supports the conclusions. Experiments must have been conducted rigorously, with appropriate controls, replication, and sample sizes. The conclusions must be drawn appropriately based on the data presented. 

Reviewer #2: Yes

Reviewer #3: Yes

**********

3. Has the statistical analysis been performed appropriately and rigorously? 

Reviewer #2: N/A

Reviewer #3: N/A

**********

4. Have the authors made all data underlying the findings in their manuscript fully available?

The PLOS Data policy requires authors to make all data underlying the findings described in their manuscript fully available without restriction, with rare exception (please refer to the Data Availability Statement in the manuscript PDF file). The data should be provided as part of the manuscript or its supporting information, or deposited to a public repository. For example, in addition to summary statistics, the data points behind means, medians and variance measures should be available. If there are restrictions on publicly sharing data—e.g. participant privacy or use of data from a third party—those must be specified.

Reviewer #2: Yes

Reviewer #3: No

**********

5. Is the manuscript presented in an intelligible fashion and written in standard English?

PLOS ONE does not copyedit accepted manuscripts, so the language in submitted articles must be clear, correct, and unambiguous. Any typographical or grammatical errors should be corrected at revision, so please note any specific errors here.

Reviewer #2: Yes

Reviewer #3: Yes

**********

6. Review Comments to the Author

Please use the space provided to explain your answers to the questions above. You may also include additional comments for the author, including concerns about dual publication, research ethics, or publication ethics. (Please upload your review as an attachment if it exceeds 20,000 characters)

Reviewer #2: The manuscript has improved since the previous version and the authors have answered all my questions.

Reviewer #3: - The data analysis lacks a section about the different aspects of trustworthiness of the study. In the discussion, the internal validity is addressed to some extent, but the manuscript lacks aspects of credibility, transferability, dependability, and confirmability.

- Related to trustworthiness of the study: how were the authors IM and MH (in addition to the main researchers WS, KA and MdJ) involved in conforming the themes identified in the study?

- I could not find a statement about availability of data.

**********

7. PLOS authors have the option to publish the peer review history of their article (what does this mean?). If published, this will include your full peer review and any attached files.

If you choose “no”, your identity will remain anonymous but your review may still be made public.

**Do you want your identity to be public for this peer review?** For information about this choice, including consent withdrawal, please see our Privacy Policy.

Reviewer #2: No

Reviewer #3: No

**********

[NOTE: If reviewer comments were submitted as an attachment file, they will be attached to this email and accessible via the submission site. Please log into your account, locate the manuscript record, and check for the action link "View Attachments". If this link does not appear, there are no attachment files.]

While revising your submission, please upload your figure files to the Preflight Analysis and Conversion Engine (PACE) digital diagnostic tool, https://pacev2.apexcovantage.com/. PACE helps ensure that figures meet PLOS requirements. To use PACE, you must first register as a user. Registration is free. Then, login and navigate to the UPLOAD tab, where you will find detailed instructions on how to use the tool. If you encounter any issues or have any questions when using PACE, please email PLOS at figures@plos.org. Please note that Supporting Information files do not need this step.

# Author comment: Author response to Decision Letter 1

**DOI:** 10.1371/journal.pone.0285822.r004

17 Jan 2023

We refer to our response to the reviewers.

Attachment Submitted filename: Response to reviewers revised manuscript 2023.docx Click here for additional data file.

# Peer review: Decision Letter 2

**DOI:** 10.1371/journal.pone.0285822.r005

**Authors:** Sabina De Rosis

3 May 2023

Facing the challenges of PROM implementation in Dutch dialysis care: patients’ and professionals’ perspectives

PONE-D-21-17498R2

Dear Dr. Sipma,

We’re pleased to inform you that your manuscript has been judged scientifically suitable for publication and will be formally accepted for publication once it meets all outstanding technical requirements.

Within one week, you’ll receive an e-mail detailing the required amendments. When these have been addressed, you’ll receive a formal acceptance letter and your manuscript will be scheduled for publication.

An invoice for payment will follow shortly after the formal acceptance. To ensure an efficient process, please log into Editorial Manager at http://www.editorialmanager.com/pone/, click the 'Update My Information' link at the top of the page, and double check that your user information is up-to-date. If you have any billing related questions, please contact our Author Billing department directly at authorbilling@plos.org.

If your institution or institutions have a press office, please notify them about your upcoming paper to help maximize its impact. If they’ll be preparing press materials, please inform our press team as soon as possible -- no later than 48 hours after receiving the formal acceptance. Your manuscript will remain under strict press embargo until 2 pm Eastern Time on the date of publication. For more information, please contact onepress@plos.org.

Kind regards,

Sabina De Rosis, PhD

Academic Editor

PLOS ONE

Additional Editor Comments (optional):

Reviewers' comments:

Reviewer's Responses to Questions

**Comments to the Author**

1. If the authors have adequately addressed your comments raised in a previous round of review and you feel that this manuscript is now acceptable for publication, you may indicate that here to bypass the “Comments to the Author” section, enter your conflict of interest statement in the “Confidential to Editor” section, and submit your "Accept" recommendation.

Reviewer #2: All comments have been addressed

Reviewer #3: All comments have been addressed

**********

2. Is the manuscript technically sound, and do the data support the conclusions?

The manuscript must describe a technically sound piece of scientific research with data that supports the conclusions. Experiments must have been conducted rigorously, with appropriate controls, replication, and sample sizes. The conclusions must be drawn appropriately based on the data presented. 

Reviewer #2: (No Response)

Reviewer #3: Yes

**********

3. Has the statistical analysis been performed appropriately and rigorously? 

Reviewer #2: (No Response)

Reviewer #3: N/A

**********

4. Have the authors made all data underlying the findings in their manuscript fully available?

The PLOS Data policy requires authors to make all data underlying the findings described in their manuscript fully available without restriction, with rare exception (please refer to the Data Availability Statement in the manuscript PDF file). The data should be provided as part of the manuscript or its supporting information, or deposited to a public repository. For example, in addition to summary statistics, the data points behind means, medians and variance measures should be available. If there are restrictions on publicly sharing data—e.g. participant privacy or use of data from a third party—those must be specified.

Reviewer #2: (No Response)

Reviewer #3: Yes

**********

5. Is the manuscript presented in an intelligible fashion and written in standard English?

PLOS ONE does not copyedit accepted manuscripts, so the language in submitted articles must be clear, correct, and unambiguous. Any typographical or grammatical errors should be corrected at revision, so please note any specific errors here.

Reviewer #2: (No Response)

Reviewer #3: Yes

**********

6. Review Comments to the Author

Please use the space provided to explain your answers to the questions above. You may also include additional comments for the author, including concerns about dual publication, research ethics, or publication ethics. (Please upload your review as an attachment if it exceeds 20,000 characters)

Reviewer #2: (No Response)

Reviewer #3: (No Response)

**********

7. PLOS authors have the option to publish the peer review history of their article (what does this mean?). If published, this will include your full peer review and any attached files.

If you choose “no”, your identity will remain anonymous but your review may still be made public.

**Do you want your identity to be public for this peer review?** For information about this choice, including consent withdrawal, please see our Privacy Policy.

Reviewer #2: No

Reviewer #3: No

**********

# Editor report: Acceptance letter

**DOI:** 10.1371/journal.pone.0285822.r006

**Authors:** Sabina De Rosis

5 May 2023

PONE-D-21-17498R2 

Facing the challenges of PROM implementation in Dutch dialysis care: patients’ and professionals’ perspectives 

Dear Dr. Sipma:

I'm pleased to inform you that your manuscript has been deemed suitable for publication in PLOS ONE. Congratulations! Your manuscript is now with our production department. 

If your institution or institutions have a press office, please let them know about your upcoming paper now to help maximize its impact. If they'll be preparing press materials, please inform our press team within the next 48 hours. Your manuscript will remain under strict press embargo until 2 pm Eastern Time on the date of publication. For more information please contact onepress@plos.org.

If we can help with anything else, please email us at plosone@plos.org. 

Thank you for submitting your work to PLOS ONE and supporting open access. 

Kind regards, 

PLOS ONE Editorial Office Staff

on behalf of

Dr. Sabina De Rosis 

Academic Editor

PLOS ONE
